# A proteolytic nanobiocatalyst with built-in disulphide reducing properties†

**DOI:** 10.1039/d0ra10013g

**Published:** 2020-12-24

**Authors:** Manon L. Briand, Maria Bikaki, Chasper Puorger, Philippe F.-X. Corvini, Patrick Shahgaldian

**Affiliations:** School of Life Science, University of Applied Sciences and Arts Northwestern Switzerland Hofackerstrasse 30, Muttenz CH-4132 Switzerland patrick.shahgaldian@fhnw.ch

## Abstract

We report a method to equip proteolytic nanobiocatalysts with intrinsic disulphide bond reducing properties. After immobilisation onto silica particles, selected protease enzymes are partially shielded in a nanometre-thick mercaptosilica layer acting not only as a protective system but also as a substrate reducing agent. The biocatalysts produced efficiently perform simultaneous disulphide bond reduction and protein digestion. Besides a significant simplification of the proteolysis process, this strategy allows for a drastic increase of the enzyme stability.

## Abstract 2

Proteolytic nanobiocatalysts are equipped with intrinsic disulphide bond reducing properties.

## Graphical abstract

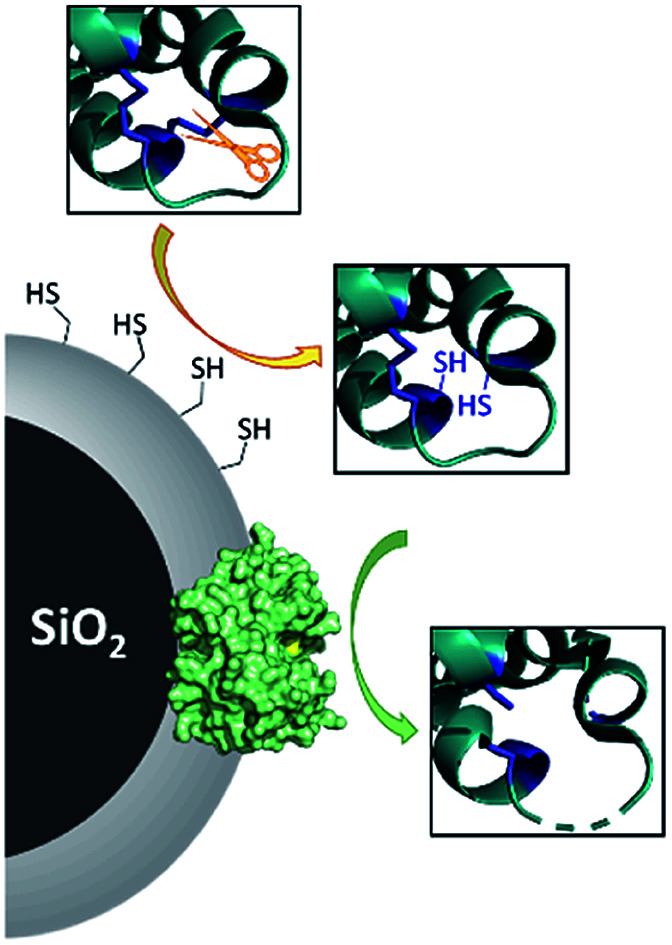

## Introduction

Owing to their remarkable proteolytic activities, proteases are widely used in a number of industrial sectors including food and feed, waste management and detergent.^1,2^ Specialised proteases (*e.g.*, Trypsin) are also of great importance in proteomics and molecular biology.^3,4^ The use of soluble proteases is, however, often hindered by their limited recovery and reusability after the biochemical reaction as well as their susceptibility to autolysis.^5^ The immobilisation of proteases at the surface of solid carrier materials allows circumventing these issues and is commonly considered as a valuable alternative to the use of soluble proteases.^6,7^ In the field of proteomics, peptide mapping for protein identification often includes protein digestion with multiple enzymes. The peptides recovered are analysed by liquid chromatography – high resolution mass spectrometry (LC-MS) and the results obtained are further compared with protein databases.^8,9^ Simple sample buffers or volatile compounds are compatible to LC-MS instrumentation. However, very frequently, more complex buffers are needed for an effective enzymatic digestion. For example, protein unfolding prior to digestion is an essential step for a successful hydrolysis. For this purpose, chemical reagents, such as urea, mercaptoethanol, dithiothreitol (DTT) or sodium dodecyl sulphate (SDS) are commonly used.^10–13^ Those treatments typically require additional clean-up step prior to LC-MS analysis, which might entail peptide losses.^14^ The presence of non-volatile solvents or buffers results in inadequate ionisation in the source of the mass spectrometer negatively affecting the ionisation efficiency. Consequently, mass ion suppression might lead to erroneous results.^15^

We have previously introduced a synthetic chemical strategy to fully shield immobilised enzymes in an organosilica layer at the surface of silica particles (SPs).^16,17^ We demonstrated that this organosilica layer protects the enzyme in a soft environment enhancing enzyme stability while maintaining catalytic activity. However, fully shielded enzymes can only convert small substrates, which can diffuse through the protective layer. To tackle this limitation, we have developed a method allowing partial chemical encapsulation and protection of enzymes processing large protein substrates.^18^ This method allows the partial shielding of enzymes in an organosilica layer of controlled thickness while maintaining the access of the protein substrates to the enzyme active site for the biocatalytic reaction. This partial shield was demonstrated to drastically stabilise immobilised enzymes. However, when it comes to proteolysis (*e.g.*, for food or pharmaceutical applications), the cross-contamination of the final product with the chemical reagents needed for protein unfolding remains a major issue and new solutions are needed.

We propose and describe, herein, a method allowing the partial shielding of immobilised proteases in a layer equipped with disulphide reducing properties. The resulting nanobiocatalyst is assessed for its ability to simultaneously reduce and digest proteins and is expected to be used in applications for which the contamination of the reaction product is problematic.

## Results and discussion

**Fig. 1:**
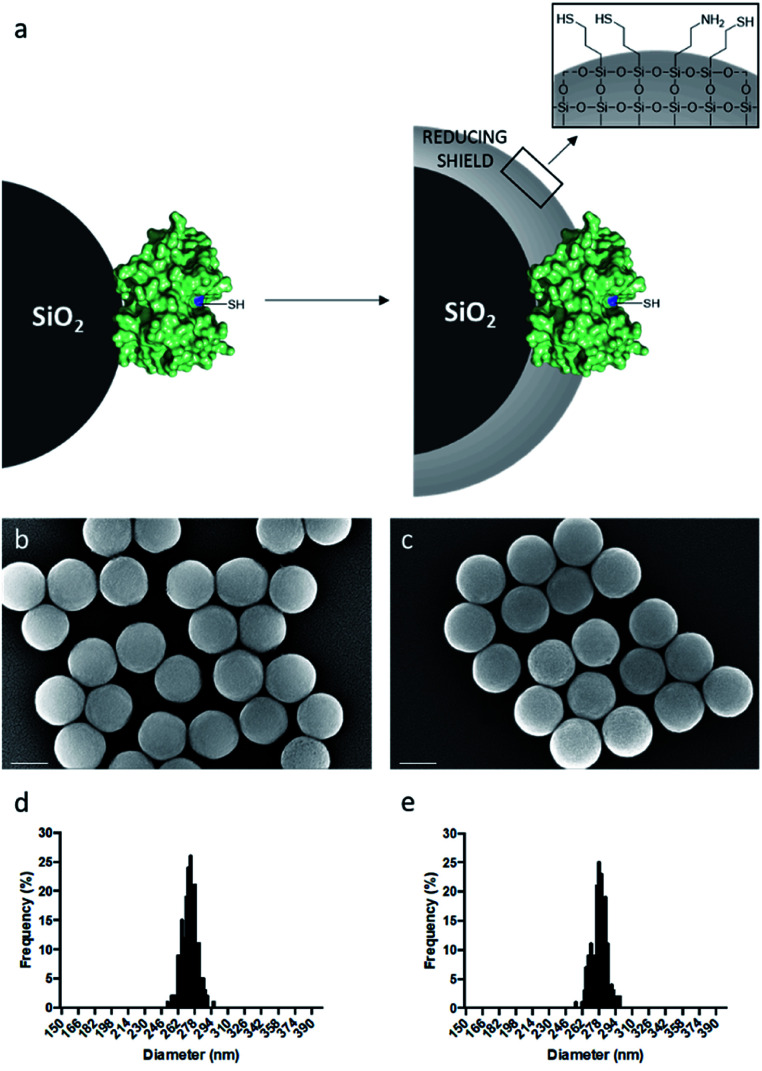
Protection of papain in a thin mercaptosilica layer. (a) Scheme of the partial shielding of papain in a reducing layer made of A, T and S; SEM micrographs of (b) bare SPs and (c) SPs-Pap-ATS (10*X*). Scale bars represent 200 nm. Statistical analysis of SEM micrographs of (d) bare SPs and (e) SPs-Pap-ATS (10*X*).

**Fig. 2:**
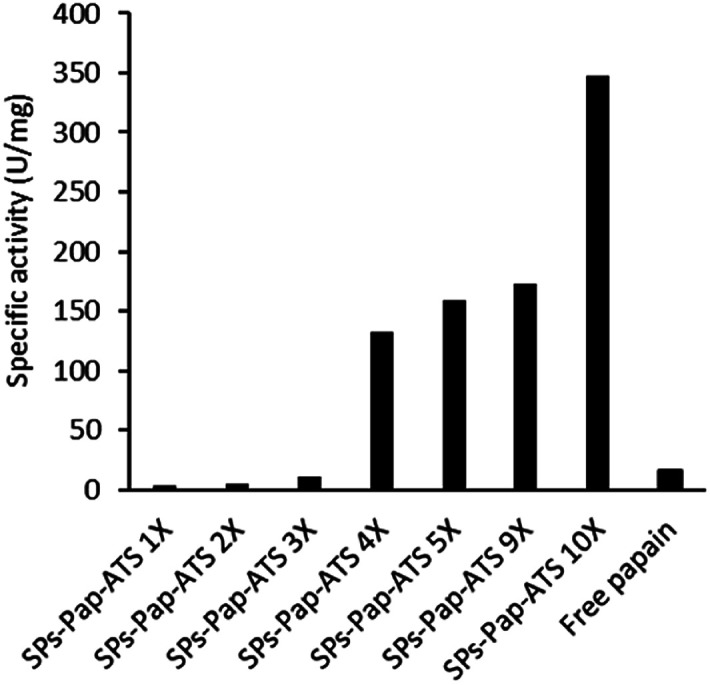
Influence of the mercaptosilane concentration on the specific activity of immobilised papain.

As model enzyme, we used papain (Pap), a protease that possesses a nucleophilic cysteine thiol in a catalytic diad.^19^ The immobilisation of papain on silica particles (SPs) and the partial shielding of this enzyme in a thin organosilica layer were performed. SPs were produced following a method previously reported.^20^ Amino-modification of the SPs surface was performed using (3-aminopropyl)triethoxysilane (A) to yield SPs-NH_2_. Papain was immobilised on SPs-NH_2_*via* imine bond formation of the crosslinker, namely glutaraldehyde, with amino residues of SP-NH_2_ and lysines of the protein to yield SPs-Pap. Protein quantification assays performed on the reaction supernatant showed immobilisation of 8.6 μg of papain per mg of SPs-NH_2_, corresponding to a coverage of *ca.* 50% of the available SPs surface (Table S1†). Further, the growth of a partial organosilica layer aiming at protecting the protease was performed. Papain is a monomeric enzyme with an estimated diameter of 4.5 nm, based on protein structural analysis (PDB code: 1PPN).^21^ Thus, we aimed at producing an organosilica layer not exceeding *ca.* 2.5 nm to maintain the active site accessible to large substrates. In order to endow the nanobiocatalysts produced with cystine-reducing properties, we decided to protect and stabilise papain in a mercaptosilica layer. To that end, SPs-Pap was incubated with tetra-ethyl-orthosilicate (T), A and 3-mercaptopropyltriethoxysilane (S) to yield the biocatalyst SPs-Pap-ATS (Fig. 1a). As reference material, thiolated particles SPs-ATS were produced by incubation of SPs-NH_2_ with A, T and S, omitting the enzyme (see below). In order to determine the effect of the thiol density on papain activity, thiolated layers were grown at the surface of SPs-Pap using increasing concentrations of S (1*X*, 2*X*, 3*X*, 4*X*, 5*X*, 9*X*, 10*X*; where *X* represents the concentration factor and 1*X* is the equivalent of 7.6 mM; Table S2†). In order to verify that the layer was thin enough to allow large protein substrates to reach the immobilised papain active site, a universal protease assay using casein as substrate was applied (Fig. 2).^22^ The results obtained clearly showed that, despite the presence of the protective layer, conversion of a substrate as large as casein occurred. The activities of the different nanobiocatalysts were compared to the activity of the soluble enzyme. Papain activity is improved with increasing mercaptosilane concentration to reach a specific activity of 348 U mg^−1^ when using 10*X* mercaptosilane S. Remarkably, SPs-Pap-ATS (10*X*) is twenty times more active than soluble papain.

To better understand this remarkable increase of the proteolytic activity of the partially shielded enzyme, we selected SPs-Pap-ATS (10*X*) for further characterisation and digestion experiments. SPs-Pap-ATS (10*X*) was also imaged by scanning electron microscopy (SEM) and compared to the bare SPs by statistical analysis of scanning electron micrographs (Fig. 1b and c). The surfaces of both bare SPs and SPs-Pap-ATS (10*X*) appear fairly smooth. As expected, an increase of the particle diameter was observed (Fig. 1d and e, Table S3†) and a layer thickness of 2.7 nm was measured at the surface of SPs-Pap-ATS (10*X*).

In order to confirm the disulphide reducing properties of SPs-Pap-ATS (10*X*), we incubated the reference material SPs-ATS with bovine serum albumin (BSA). We expected these particles to act as disulphide reducing agent facilitating protein unfolding. BSA was selected to facilitate the data interpretation after the biochemical reaction.^23^ In order to assess the effect of the treatment on the target protein folding, tryptophan (Trp) fluorescence emission spectra of both native BSA and BSA after incubation with SPs-ATS were performed. Measurement were carried out at an excitation wavelength of 295 nm (Fig. S1†) in order to minimise tyrosine excitation and thus only observe the effect of the environment on the tryptophan residues.^24^ BSA incubated with thiolated particles showed a significantly lower intrinsic fluorescence than the native protein. This decrease in fluorescence is explained by a higher exposition of Trp residues to the buffer, demonstrating a change in the protein folding state. In order to quantify the disulphide bond reduction caused by SPs-ATS, Ellman's reaction was performed on the reaction supernatant (Table S7†).^25^ As a control experiment, BSA was reduced using DTT as reducing agent. The thiol content of reduced BSA was compared to that of BSA in the fully reduced state after DTT treatment. Native BSA possesses 17 disulphide bonds and one free cysteine. After incubation of BSA with SPs-ATS, an average of 11 disulphide bonds per BSA molecule were cleaved, demonstrating the disulphide reducing power of the thiolated particles. This incomplete reduction was attributed to the lack of accessibility of disulphide bonds buried in the three dimensional structure of BSA.^26^

**Fig. 3:**
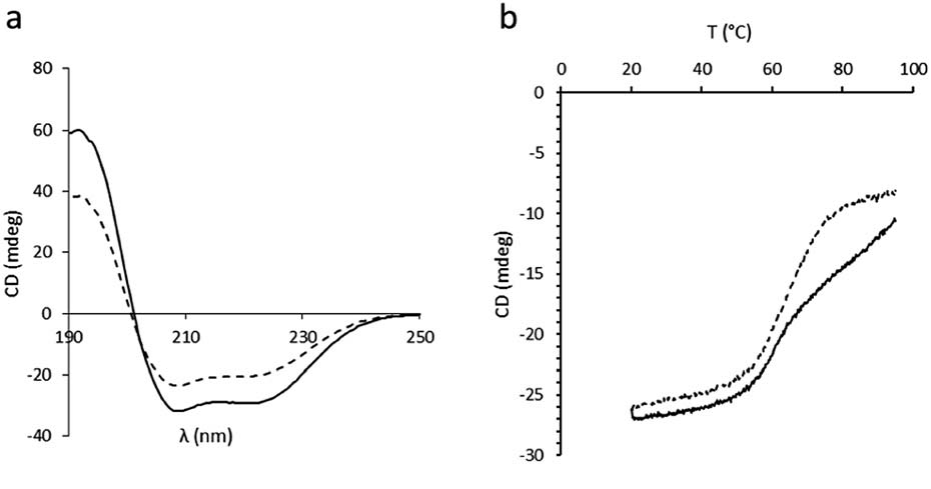
CD experiments. (a) CD spectra at 25 °C and (b) CD melting curves at 208 nm of BSA (solid line) and BSA after incubation with SPs-ATS (dashed line).

In order to assess the impact of the thiolated particles on the secondary structure of BSA, circular dichroism (CD) experiments on both native BSA and BSA after incubation with SPs-ATS were carried out at 25 °C (Fig. 3a). A loss in CD signal after incubation with the thiolated particles, characterised by a decreased ellipticity, was observed, indicating a change of the secondary structure of the protein. This decrease of ellipticity was attributed to a diminution of the initial α-helix content of BSA after incubation with SPs-ATS.^27^ To rule out the possibility of non-specific adsorption of BSA at the surface of SPs-ATS, which would lead to a decrease of BSA concentration and could explain these changes of spectroscopic parameters, a protein quantification assay was performed on the reaction supernatant (Tables S4 and S5†). No change in BSA concentration after incubation with SPs-ATS was measured. Then, CD melting curves of both native BSA and BSA incubated with SPs-ATS were performed from 20 to 95 °C at 208 nm (Fig. 3b). Important differences were observed between the two melting curves. Native BSA appeared to have two transitions, one at *ca.* 58 °C and the other one at *ca.* 75 °C, consistent with previously reported results.^28^ In the case of BSA incubated with SPs-ATS, the second transition at higher temperature is not measured, confirming the partial destabilisation of the protein structure. Melting temperatures (*T*_m_) and enthalpy changes for unfolding at *T*_m_ (Δ*H*_m_) were calculated using the thermal denaturation equation (Fig. S2, Table S6†).^29^ A change of Δ*H*_m_ (from 24 kcal mol^−1^ for native BSA to 17.9 kcal mol^−1^ for BSA after incubation with SPs-ATS) and *T*_m_ (from 58 °C for native BSA to 65 °C for BSA after incubation with SPs-ATS) were measured. These changes of Δ*H*_m_ and *T*_m_ were attributed to the different behaviours of native BSA, which shows two transitions, and BSA after incubation with SPs-ATS, which exhibits only one broader transition. These differences of CD spectra and melting curves demonstrated a change of BSA secondary structure after incubation with SPs-ATS, attributed to the reducing capacity of the thiolated particles.

In order to assess further the proteolytic activity of the biocatalysts produced, we used SPs-Pap-ATS (10*X*) for BSA proteolysis. BSA was incubated with SPs-Pap-ATS (10*X*) at 37 °C in phosphate buffer (10 mM, pH 7) for 18 hours. The recovered peptides were analysed by LC-MS/MS and compared with those obtained after BSA digestion with soluble papain without prior reduction of BSA (Fig. S6 and S7†). BSA digested with SPs-Pap-ATS (10*X*) generated a more complex chromatogram with regard to that obtained with soluble papain, which indicates the formation of a higher number of peptides. The results were further analysed using the Spectrum Mill software for peptide and protein identification. BSA digested with SPs-Pap-ATS (10*X*) generated 106 identified peptides (Table S10†) while soluble papain, without prior reduction of the protein to digest, generated only 13 peptides (Table S11†). Cleavage specificity was assessed by analysing the residues in C-terminal positions of the peptides produced (Table 1). In both cases, the data obtained showed a preference of cleavage at the C-terminus of lysine with 31% and 26% of the cleavages on this position with the soluble enzyme and SPs-Pap-ATS, respectively. However, a broadening of substrate selectivity is observed with SPs-Pap-ATS (10*X*). Interestingly, cleavages at the C-terminus of hydrophobic amino acids such as alanine, methionine and valine (which are usually buried inside the protein core) are also observed. One can safely postulate that these hydrophobic residues became available for cleavage owing to substrate unfolding attributed to the reductive effect provided by the thiolated environment of papain. As control experiment, we performed BSA digestion with soluble papain after incubation of BSA with SPs-ATS (Table 1, Fig. S9†) or after BSA unfolding by heating (Fig. S8†). Similar peptide recovery and cleavage sites than those obtained after incubation with SPs-Pap-ATS (10*X*) were obtained.

**Table 1:** Residues present at C-term end (%) of digested BSA after treatment with soluble papain, SPs-Pap-ATS (10*X*) and soluble papain after disulphide reduction using SPs-ATS

	Soluble papain	SPs-Pap-ATS (10*X*)	SPs-ATS + soluble papain
Lysine (K)	31	26	29
Leucine (L)	8	8	10
Methionine (M)	—	1	—
Asparagine (N)	—	2	1
Glutamine (Q)	—	2	2
Arginine (R)	8	3	2
Serine (S)	—	11	7
Threonine (T)	8	6	4
Valine (V)	—	1	1
Tryptophan (W)	8	3	2
Tyrosine (Y)	—	1	3
Alanine (A)	—	8	7
Cysteine (C)	—	4	7
Aspartate (D)	—	2	2
Glutamate (E)	15	9	15
Phenylalanine (F)	7	4	4
Glycine (G)	7	5	1
Histidine (H)	8	5	2

LC-MS/MS analyses of digested casein with both soluble and immobilised papain were also performed (Fig. S10 and S11†). Here again, the most complex chromatogram was obtained after protein digestion with SPs-Pap-ATS (10*X*), thus confirming the previous observations. These results therefore confirmed our initial assumption: SPs-Pap-ATS (10*X*) allows simultaneous reduction and digestion of proteins.

To assess the stability of SPs-Pap-ATS (10*X*) and free papain, universal protease assays were performed at increasing storage durations (Fig. S12†). The soluble papain showed a drastic decrease of activity with no remaining activity after 51 days whereas SPs-Pap-ATS (10*X*) exhibited remarkable stability with as much as 70% and 56% remaining activity after 51 days and 142 days of storage, respectively. This result clearly confirmed the protective effect of the partial mercaptosilica shield on the immobilised protease. Moreover, it proved that this reducing mercaptosilica layer did not lead to the reduction and unfolding of the immobilised protease.

In order to gather additional insights in the reduction mechanism, additional biocatalyst recycling experiments were performed. SPs-Pap-ATS (10*X*) was used to digest casein. After first use, the biocatalytic material was recovered and reused to digest casein; the results showed a consistent decrease in biocatalytic activity of 85% (Fig. S4†). In order to confirm that this loss in activity was due, as initially hypothesized, to the consumption of the thiol functions at the surface of the thiolated particles during protein unfolding, the reference material SPs-ATS was incubated with BSA. SPs-ATS were reused five times. After removal of SPs-ATS, thiol contents of both BSA and SPs-ATS were measured. A decrease of BSA thiol content after each SPs-ATS use was observed, resulting in a loss of 98% of the initial reducing capacity of the thiolated material after five uses (Fig. S3†). This is explained by the full oxidation of the thiol functions at the surface of SPs-ATS (Table S9†). Our experimental results suggest a *ca.* 5-fold excess of thiol functions compared to the BSA thiol functions (Table S8†). One can therefore safely assume that the excess of thiol functions in the protective layer allows the release of the substrate protein by intramolecular rearrangement in the protective layer. This is confirmed by the fact that there is no relevant decrease of BSA concentration after reduction with SPs-ATS (Tables S4 and S5†). Thus, the intramolecular rearrangement in the protective layer results in the formation of intramolecular disulphide bridges, leading to the oxidative saturation of the thiolated particles.

To demonstrate the method versatility, the strategy was applied to another protease, namely Subtilisin A (SubA).^30^ SPs-SubA-ATS (10*X*) was prepared as described above (see ESI for details, Table S12†). To first assess the proteolytic activity of the nanobiocatalyst produced, a universal protease assay was performed (Fig. S13†). The specific activity of SPs-SubA-ATS (10*X*) on casein substrate was compared to the one of soluble SubA and showed similar results with a specific activity of around 210 U mg^−1^ in both cases. Then, SPs-SubA-ATS (10*X*) was used for digesting BSA. The digested peptide fragments were analysed by LC-MS/MS and compared with those obtained after BSA digestion with soluble SubA without prior BSA reduction with a reducing agent (Fig. S14†). As for Pap, the BSA digestion performed with SPs-SubA-ATS (10*X*) generated a more complex base peak chromatogram than soluble SubA indicating a higher peptide recovery compared to free SubA. Moreover, 275 peptides were identified after BSA digestion with SPs-SubA-ATS (10*X*), while soluble SubA allowed the identification of only 59 peptides (Tables S13 and S14†). This set of results confirm the versatility of our method.

## Experimental section

### Materials and methods

#### Chemicals

Tetraethyl orthosilicate (T, ≥ 99%), (3-aminopropyl)triethoxysilane (A, ≥ 98%), glutaraldehyde (Grade I, 50% in H_2_O), casein from bovine milk, bovine serum albumin (BSA, ≥ 96%), Folin & Ciocalteu's phenol reagent (2N), 5-5′-dithiobis(2-nitrobenzoic acid) (Ellman's reagent, ≥ 98%), trichloroacetic acid solution (TCA, 6.1 N), sodium carbonate (Na_2_CO_3_, ≥ 99.5%), papain from Papaya Latex (1.5–10 units per mg solid) and subtilisin A (protease from Bacillus licheniformis) were purchased from Sigma-Aldrich (Switzerland). Potassium phosphate salts were purchased from Fisher Scientific (Switzerland). 3-Mercaptopropyltriethoxysilane (S, 98%) was purchased from ABCR (Germany). Bradford protein Assay was purchased form Bio-Rad (Switzerland).

#### Equipment

Universal protease assays were performed in a Thermomixer (Thermomixer Comfort, Eppendorf, Germany). Tryptophan fluorescence was monitored using a Synergy H1 (BioTek, Switzerland) in 96-well plate (Microplate 96 Well Half Area, Huber Lab, Switzerland). Circular dichroism measurements were performed with a JASCO J-810 CD spectropolarimeter in an absorption cuvette of quartz suprasil with a path length of 1 mm (VWR, Switzerland). Nanopure water (resistivity ≥ 18 MΩ-cm) was produced with a Millipore ®Synergy purification system (Merck, Switzerland). Particles were imaged using a Zeiss SUPRA® 40VP scanning electron microscope (Germany). Particle sizes were measured using the ®AnalySIS software package.

### Experimental procedures

#### Papain immobilisation

To a suspension of SPs-NH_2_ (5.1 mL, 8.7 mg mL^−1^) was added glutaraldehyde (29 μL). The reaction mixture was stirred at 20 °C, 400 rpm for 30 min. Then, the suspension was washed twice in H_2_O, sonicated and resuspended in 2.335 mL of phosphate buffer (10 mM, pH 8) to yield SPs-GA (19 mg mL^−1^).

To SPs-GA (2.775 mL, 16 mg mL^−1^) in phosphate buffer (10 mM, pH 8) was added Papain (final concentration of 200 μg mL^−1^). The resulting suspension was stirred at 20 °C, 400 rpm for 1 h to produce SPs-Pap. A Bradford assay was performed on the supernatant collected after protein immobilisation and showed that 138 μg mL^−1^ of the enzyme was immobilised at the surface of SPs-GA.

#### Papain protection

Different layer growth conditions were tested. In a typical layer growth experiment, SPs-Pap (5 mL, 3.2 mg mL^−1^) in phosphate buffer (10 mM, pH 8) was incubated with T (14.9 μL, 0.067 mmol) at 10 °C, 400 rpm for 1 h. Subsequently, A (5.7 μL, 0.024 mmol) and S (see ESI† for details) were added to the reaction media. The resulting mixture was stirred at 10 °C, 400 rpm for 1 h to yield SPs-Pap-ATS. The particles were centrifuged at 350 rcf for 5 min, washed three times in phosphate buffer (10 mM, pH 8) and resuspended in 1 mL phosphate buffer ([SPs-Pap-ATS] = 16 mg mL^−1^). The biocatalysts produced were allowed to cure at room temperature for 12 hours and finally stored at 4 °C.

#### Scanning electron microscopy characterisation

Solutions of silica particles in nanopure water were prepared and a volume of 2 μL was spread on a freshly cleaned silicon wafer. The different samples were dried and sputter-coated with a gold–platinum alloy at 20 mA for 15 s. Scanning electron micrographs were acquired using the InLens mode at a magnification of 150 000× with an accelerating voltage of 10 kV. Particle sizes were measured using the ®AnalySIS software package. At least 100 measurements were performed for each sample.

#### Preparation of SPs-ATS

A suspension of SPs-NH_2_ (5 mL, 3.2 mg mL^−1^) in phosphate buffer (10 mM, pH 8) was incubated with T (14.9 μL) at 10 °C, 400 rpm for 1 h. Subsequently, A (5.69 μL) and S (90.9 μL) were added to the reaction media. The resulting mixture was stirred at 10 °C, 400 rpm for 1 h to yield SPs-ATS. The particles were centrifuged at 4000 rpm for 5 min, washed three times in phosphate buffer (10 mM, pH 8) and resuspended in 1 mL phosphate buffer ([SPs-ATS] = 16 mg mL^−1^). The particles produced were allowed to cure at room temperature for 12 hours and finally stored at 4 °C.

#### Reduction of BSA with SPs-ATS

SPs-ATS (60 μL) in phosphate buffer (10 mM, pH 7) was added to a solution of BSA (240 μL) in phosphate buffer (10 mM, pH 7) to reach a final BSA concentration of 1 mg mL^−1^. As control experiment, BSA was reduced using DTT as reducing agent. The resulting mixture was stirred at 37 °C, 750 rpm for 1 hour. The particles were centrifuged and the supernatant containing the reaction products was collected. In the control experiment, DTT was removed by centrifugation using Amicon® Ultra Centrifugal Filters. A negative control experiment using native BSA was performed. Supernatants were used for protein quantification assay (Bradford, Tables S4 and S5†), steady-state fluorescence measurements (Fig. S1†), for determining the thiol content of BSA after incubation with SPs-ATS (Ellman's reaction, Table S7†) and for CD experiments (Fig. S2,† Table S6†).

The recyclability of SPs-ATS was tested by repetitive incubation of the same batch of particles with BSA and determination of the thiol content of BSA after incubation with the reused SPs-ATS (Fig. S3†).

#### CD experiments

Supernatants were diluted 5 times ([BSA] = 200 μg mL^−1^) for CD analysis. CD experiments on both native BSA and BSA after incubation with SPs-ATS were carried out at 25 °C, from 250 nm to 190 nm in an absorption cuvette.

#### Melting curves

Supernatants were diluted 5 times ([BSA] = 200 μg mL^−1^). Melting curves of both native BSA and BSA incubated with SPs-ATS were performed from 20 to 95 °C at 208 nm in an absorption cuvette with a pathlength of 1 mm.

#### Ellman's reaction

Ellman's reagent solution was prepared by dissolving 4 mg of Ellman's reagent in 1 mL of sodium phosphate buffer (0.1 M, pH 8, 1 mM EDTA).

25 μL of each reduced sample was diluted in 250 μL of sodium phosphate buffer (0.1 M, pH 8, 1 mM EDTA). 5 μL of the Ellman's reagent solution was added to each vial. The resulting mixture was stirred at 25 °C, 750 rpm for 15 min. Absorbance was measured at 412 nm and compared to a standard curve prepared with cysteine.

#### Universal protease assay of SPs-Pap-ATS (10*X*)

After curing, immobilised and protected papain (25 μL) in phosphate buffer (50 mM, pH 7) was added to a solution of casein (125 μL, 0.65% w/v) in phosphate buffer (50 mM, pH 7). The resulting mixture was stirred at 37 °C, 750 rpm for 30 minutes. The particles were centrifuged and the supernatant containing the reaction products was collected. To the supernatant (100 μL) was added a solution of TCA (110 mM, 83.3 μL) in H_2_O, leading to the precipitation of the undigested casein. The resulting mixture was stirred at 37 °C, 750 rpm for 30 minutes. The resulting solution was centrifuged and the supernatant containing the digested fragments of casein was collected. To this supernatant (100 μL) was added a solution of sodium carbonate (500 mM, 250 μL) followed by Folin–Ciocalteu reagent (0.5 M, 50 μL). The resulting mixture was stirred at 37 °C, 750 rpm for 30 minutes. Absorbance was measured at 660 nm, on 200 μL of the resulting solutions.

The recyclability of SPs-Pap-ATS (10*X*) was tested by repetitive casein digestions using the same batch of biocatalyst, following the procedure described above (Fig. S4†).

#### BSA digestion with SPs-Pap-ATS

BSA (1 mg) was dissolved in 1 mL of phosphate buffer (10 mM, pH 7). Immobilised and non-immobilised papain were added to the BSA solution (100 μg of BSA for 1 μg of enzyme). The samples were incubated for 18 h at 37 °C. The enzymatic digestion was stopped by centrifuging the samples containing the immobilised papain at 3500 rpm at 4 °C for 5 min, whereas in the case of the samples containing the non-immobilised papain, the pH was decreased to 2 by adding formic acid. The samples were collected and injected to LC-MS instrumentation for further analyses.

#### LC-MS/MS measurements

HPLC experiments were performed on an Agilent 1290 UHPLC system using a Peptide 2.7 μm C18 column (2.1 × 250 mm, 2.7 μm particle size). LC-MS conditions were optimised for the current work aiming to achieve higher peptide detection and to facilitate data analysis afterwards. The sample injection volume was 3 μL. The binary solvent system used consisted of Milli-Q water (Solvent A) and acetonitrile (Solvent B), both containing 0.1% formic acid. The solvents flow rate was kept constant at 0.200 mL min^−1^, and the column temperature was set at 40 °C. The gradient profile used was starting with 3% B, increasing to 90% B in 53 min, followed by washing with 90% B until 58.2 min, and decreasing to 3% B until 59 min. The column was re-equilibrated for the next measurement at 3% B for 10 more minutes. The effluent HPLC system was connected to a high resolution ESI-Q-TOF mass spectrometer coupled to an electrospray ionisation source (nebulizer pressure of 35 psi, dry gas flow rate of 8 L min^−1^, and dry gas temperature of 250 °C). A ramped collision energy was applied using a formula depending on the charge state of the precursor ion: (slope) × *m*/*z*/100 + (offset), where slope varied from 3.1 to 3.6 and the offset was set either to −4.8 for peptides with charge ≥3 or to 1 for peptides with charge equal to 1 and 2. All data were acquired in positive ion mode. Both full scan spectra and MS/MS data sets were recorded. The TOF analyser was calibrated using manufacturer's reference mass standards solution before each chromatographic run.

#### Protein and peptide identification

All results were analysed using ®Spectrum Mill (Agilent Technologies). The peptide search was performed using the SwissProt database, selecting the species B. Taurus.^9^ Minimum matched peak intensity was set at 30%, maximum ambiguous precursor charge to 3 and precursor mass tolerance 20 ppm. No peptide modifications were selected.

#### Stability study of SPs-Pap-ATS (10*X*)

To assess the stability of the biocatalyst, SPs-Pap-ATS (10*X*) and free papain were stored at 4 °C in phosphate buffer (10 mM, pH 8) and tested after increasing storage duration using universal protease assay (see procedure above).

#### Subtilisin A immobilisation

To SPs-GA (1.990 mL, 16 mg mL^−1^) in borate buffer (50 mM, 10 mM CaCl2, pH 8) was added SubA. The resulting suspension was stirred at 20 °C, 400 rpm for 1 h to produce SPs-SubA. A BCA assay was performed on the supernatant collected after protein immobilisation and showed that 64 μg mL^−1^ of SubA was immobilised at the surface of SPs-GA.

#### Subtilisin A protection

SPs-SubA (9 mL, 3.2 mg mL^−1^) in phosphate buffer (10 mM, pH 8) were incubated with T (26.8 μL) at 10 °C, 400 rpm for 1 h. Then, A (10.2 μL) and S (164 μL) were added to the reaction media. The resulting mixture was stirred at 10 °C, 400 rpm for 1 h to yield SPs-SubA-ATS (10*X*). The particles were centrifuged at 350 rcf for 5 min, washed three times in phosphate buffer (10 mM, pH 8) and resuspended in 1.8 mL of phosphate buffer (16 mg mL^−1^). The biocatalyst produced was allowed to cure at room temperature for 12 hours and finally stored at 4 °C.

#### Universal protease assay of SPs-SubA-ATS (10*X*)

After curing, immobilised and protected SubA (25 μL) in phosphate buffer (50 mM, pH 7) was added to a solution of casein (125 μL, 0.65% w/v) in phosphate buffer (50 mM, pH 7). The resulting mixture was stirred at 37 °C, 750 rpm for 30 minutes. The particles were centrifuged and the supernatant containing the reaction products was taken out. To the supernatant (100 μL) was added a solution of TCA (110 mM, 83.3 μL) in H_2_O, leading to the precipitation of the undigested casein. The resulting mixture was stirred at 37 °C, 750 rpm for 30 minutes. The resulting solution was centrifuged and the supernatant containing the digested fragments of casein was collected. To the supernatant (100 μL) was added a solution of sodium carbonate (500 mM, 250 μL) followed by Folin–Ciocalteu reagent (0.5 M, 50 μL). The resulting mixture was stirred at 37 °C, 750 rpm for 30 minutes. Absorbance was measured at 660 nm, on 200 μL of the resulting solutions.

## Conclusions

In summary, we demonstrated that thiolated nanobiocatalysts effectively mimic the commonly used techniques for reducing and unfolding protein molecules prior to enzymatic digestion but with the pivotal advantage that they can be removed from the final product. Proteases partially protected in a mercaptosilica layer can easily be removed from the peptide solution, avoiding cross-contaminations of the final product with both protease and reducing agent. This is a considerable asset for applications in food or pharmaceutical industries. Moreover, our strategy allows simplifying the peptide generation process enabling simultaneous reduction and digestion of proteins. The use of chemical reagents, such as urea, DTT or SDS is not required. Consequently, sample preparation before LC-MS/MS analysis is simplified and peptide losses are avoided. Remarkably, the presence of the mercaptosilica shield does not modify protease efficiency and cleavage selectivity and allows to drastically increase enzyme stability.

## Conflicts of interest

There are no conflicts to declare.

## Supplementary Material

RA-011-D0RA10013G-s001
