# Supplementary material for: A proteolytic nanobiocatalyst with built-in disulphide reducing properties
Source: RSC Adv. 2020 Dec 24;11(2):810–6. doi: 10.1039/d0ra10013g (PMC8693372; doi:10.1039/d0ra10013g)
Supplement: RA-011-D0RA10013G-s001 [file RA-011-D0RA10013G-s001.pdf]

Electronic Supplementary Material (ESI) for RSC Advances.
This journal is © The Royal Society of Chemistry 2020

Electronic Supplementary Information (ESI)

for

A Proteolytic Nanobiocatalyst with Built-in Disulphide Reducing Properties

Manon L. Briand,‡[a] Maria Bikaki,‡[a] Chasper Puorger,[a] Philippe F.-X. Corvini[a] and Patrick Shahgaldian*[a]

[a] *School of Life Science, University of Applied Sciences and Arts Northwestern Switzerland, Hofackerstrasse 30, Muttenz CH-4132, Switzerland.*

List of figures

Figure S1. Steady-state fluorescence emission spectra of native BSA and BSA after incubation with SPs-ATS. 3
Figure S2. Melting curves at 208 nm of native BSA and BSA after incubation with SPs-ATS. 3
Figure S3. Reducing capacity of SPs-ATS after repetitive uses. 4
Figure S4. Recycling experiments of SPs-Pap-ATS (10X). 4
Figure S5. BPC (base peak chromatogram) of BSA incubated at 37°C for 18 h. 5
Figure S6. Base peak chromatogram (BPC) of BSA digested with free papain. 5
Figure S7. Base peak chromatogram (BPC) of BSA digested with SPs-Pap-ATS (10X). 5
Figure S8. BPC (base peak chromatogram) of unfolded BSA (95°C for 10min) digested with free papain. 6
Figure S9. BPC (base peak chromatogram) of unfolded BSA (with SPs-ATS) digested with free papain. 6
Figure S10. Base peak chromatogram (BPC) of casein digested with free papain. 10
Figure S11. Base peak chromatogram (BPC) of casein digested with SPs-Pap-ATS (10X). 11
Figure S12. Study of the stability overtime of SPs-Pap-ATS (10X) and soluble papain. 11
Figure S13. Universal protease assay of both soluble and immobilised SubA using casein as substrate. 12
Figure S14. Base peak chromatogram (BPC) of BSA digested with free SubA and SPs-SubA-ATS (10X). 12

List of tables

Table S1. Protein quantification assay performed on reaction supernatant after papain immobilisation. 2
Table S2. Summary of layer growth conditions. 2
Table S3. Statistical analysis of SEM micrographs of bare particles and SPs-Pap-ATS 10X. 2
Table S4. Bradford native BSA (control experiment after incubation at 37 °C, 750 rpm for 1 hour). 2
Table S5. Bradford BSA after incubation with SPs-ATS (at 37 °C, 750 rpm for 1 hour). 2
Table S6. Calculated variables from melting curves analyses of native BSA and BSA after incubation with SPs-ATS. 3
Table S7. Ellman's reaction. Thiol content of BSA after incubation with SPs-ATS. 3
Table S8. Ellman's reaction. Thiol content of SPs-ATS. 4
Table S9. Ellman's reaction. Thiol content of SPs-ATS after 5 uses. 4
Table S10. Summary of identified peptide using SPs-Pap-ATS (10X). 6
Table S 11. Summary of identified peptide using free papain. 10
Table S 12. Protein quantification assay performed on reaction supernatant after subtilisin A immobilisation. 11
Table S 13. Summary of identified peptide using free SubA. 12
Table S 14. Summary of identified peptide using SPs-SubA-ATS (10X). 15

Table S1. Protein quantification assay performed on reaction supernatant after papain immobilisation. An average concentration of 138 µg/mL of immobilised papain was calculated.

OD	$[Papain]_{supernatant}$ (µg/mL)	$[Papain]_{initial}$ (µg/mL)	$[Papain]_{immobilised}$ (µg/mL)
0.0466	67	200	133
0.0429	61	200	139
0.0398	57	200	143

Table S2. Summary of layer growth conditions.

	1X	2X	3X	4X	5X	9X	10X
**T (mmol)**	0.067	0.067	0.067	0.067	0.067	0.067	0.067
**A (mmol)**	0.024	0.024	0.024	0.024	0.024	0.024	0.024
**S (mmol)**	0.038	0.076	0.114	0.152	0.190	0.342	0.380

Table S3. Statistical analysis of SEM micrographs of bare particles and SPs-Pap-ATS 10X.

	Average (nm)	Standard deviation	Standard error	Layer thickness (nm)
**Bare SPs**	273.3	7.2	0.5	0
**SPs-Pap-ATS (10X)**	278.4	7.3	0.6	2.7

Table S4. Bradford native BSA (control experiment after incubation at 37 °C, 750 rpm for 1 hour). Supernatant was diluted 10 times.

OD	[BSA]/10 (µg/mL)	Average [BSA]/10 (µg/mL)
0.1188	148.5	
0.1076	134.5	139.9
0.1094	136.7	

Table S5. Bradford BSA after incubation with SPs-ATS (at 37 °C, 750 rpm for 1 hour). Supernatant was diluted 10 times.

OD	[BSA]/10 (µg/mL)	Average [BSA]/10 (µg/mL)
0.1038	129.7	
0.1228	153.5	139.2
0.1074	134.2	

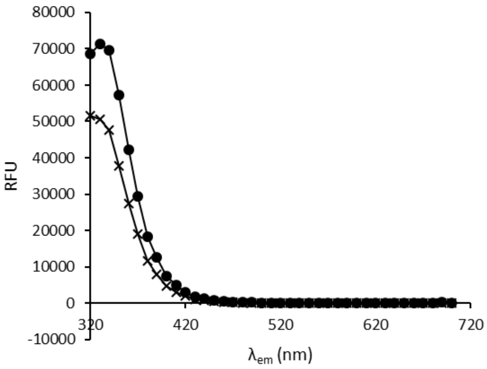

Figure S1. Steady-state fluorescence emission spectra of BSA (●) and BSA after incubation with SPs-ATS (×) in 10 mM phosphate buffer, pH 7.0. The excitation wavelength λ_{ex} was fixed at 295 nm.

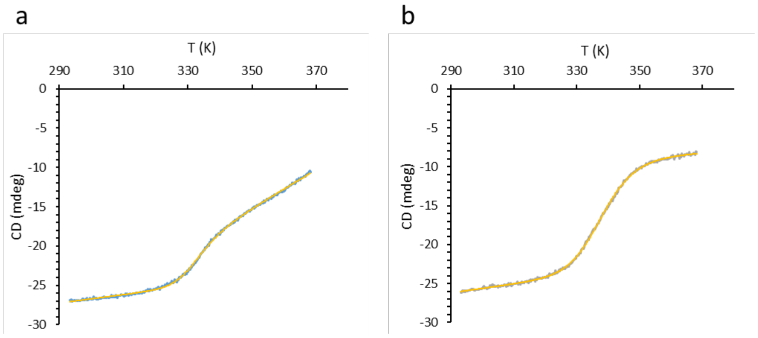

Figure S2. Melting curves at 208 nm of (a) native BSA and (b) BSA after incubation with SPs-ATS. Experimental data are shown in blue, fitted theoretical melting curves are show in yellow.

Table S6. Calculated variables from melting curves analyses of native BSA and BSA after incubation with SPs-ATS.

	s_f (mdeg)	m_f	s_u (mdeg)	m_u	ΔH_m (cal/mol)	T_m (K)
**BSA**	42.88	0.05	100.90	0.24	240137	331
**BSA + SPs-ATS**	42.60	0.06	16.38	0.02	178592	338

Table S7. Ellman's reaction. Thiol content of BSA after incubation with SPs-ATS.

	OD	[SH] (mM)	Number of SH functions	Cleaved S-S bonds
**BSA + SPs-ATS**	0.3412	0.32	24	11
**BSA + DTT**	0.5025	0.47	35	17

Table S8. Ellman's reaction. Thiol content of SPs-ATS.

OD	[SH] (mM)	Average [SH] (mM)
3.0047	2.79	
3.0455	2.83	2.80
3.0028	2.79	

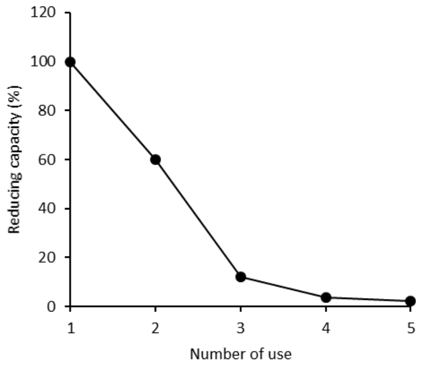

Figure S3. Reducing capacity of SPs-ATS after repetitive uses.

Table S9. Ellman's reaction. Thiol content of SPs-ATS after 5 uses.

OD	[SH] (mM)
0.0065	0.00603921

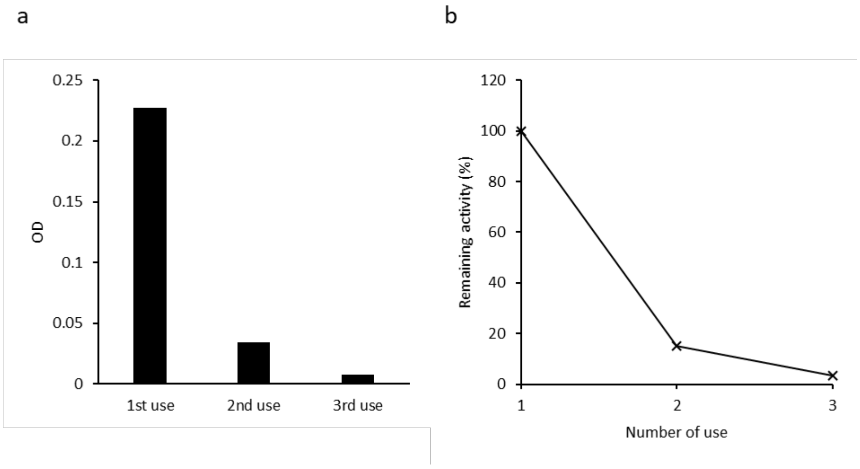

Figure S4. Recycling experiments of SPs-Pap-ATS (10X). (a) Absorbance measured in the reaction supernatant after incubation with the Folin-Ciocalteu reagent. (b) Remaining activity (%) after first, second and third use of the biocatalyst.

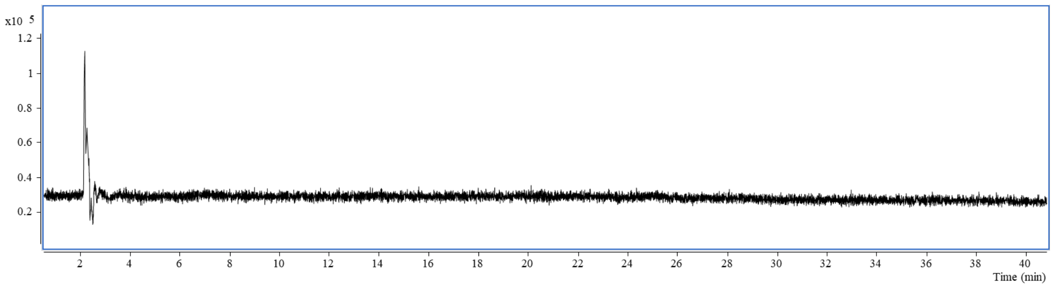

Figure S5. BPC (base peak chromatogram) of BSA incubated at 37°C for 18 h. No detectable peptides were formed.

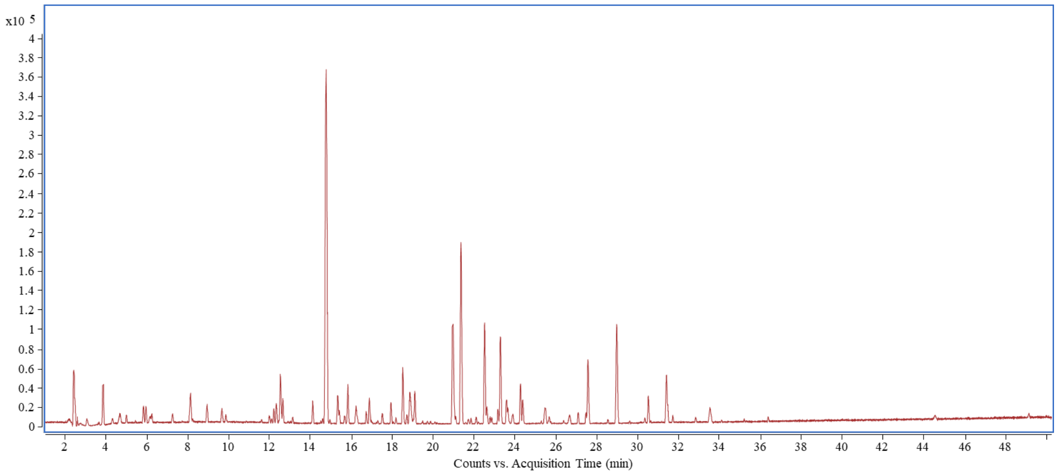

Figure S6. Base peak chromatogram (BPC) of BSA digested with free papain.

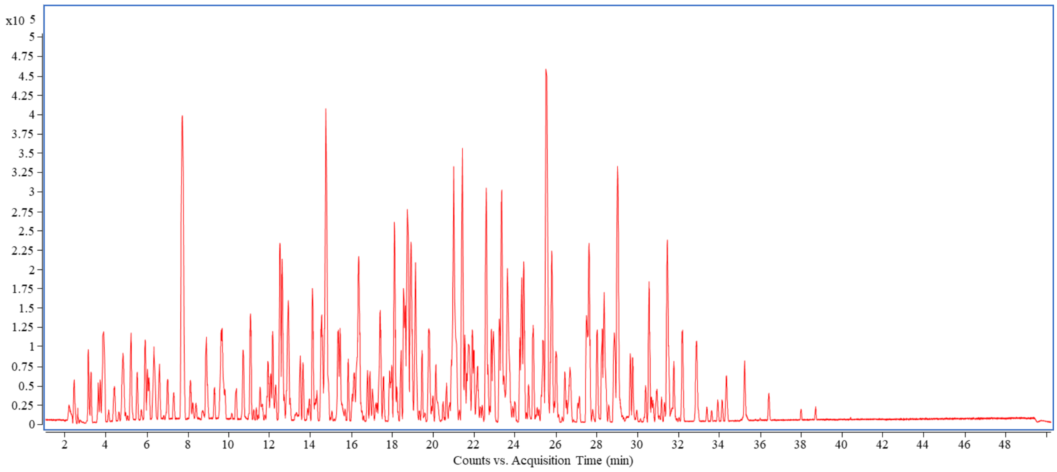

Figure S7. Base peak chromatogram (BPC) of BSA digested with SPs-Pap-ATS (10X).

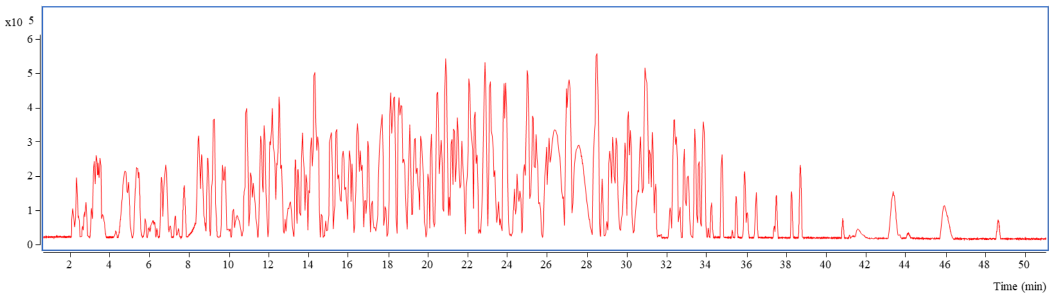

Figure S8. BPC (base peak chromatogram) of unfolded BSA (95ºC for 10min) digested with free papain.

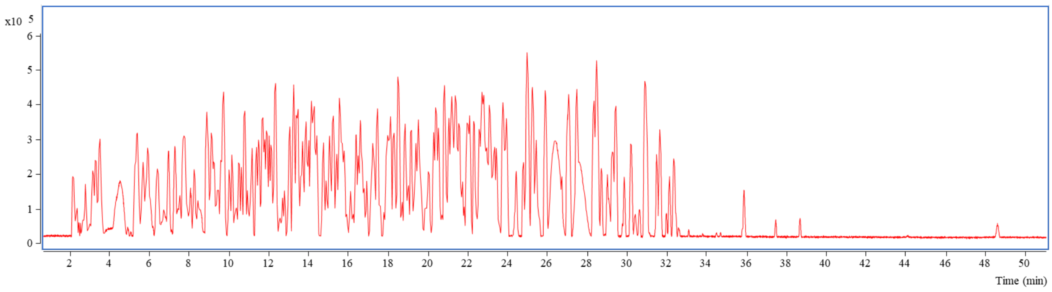

Figure S9. BPC (base peak chromatogram) of unfolded BSA (with SPs-ATS) digested with free papain.

Table S10. Summary of identified peptide using SPs-Pap-ATS (10X).

No	Precursor Charge	SPI (%)	Intensity	Sequence	RT (min)	Exp. *m/z*	Theor. mass	Error (ppm)
1	2	96.3	6.97E+05	ADFAEDKDVCK	18.68	620.782	1240.552	3.4
2	2	95.7	3.05E+05	LVTDLTKVH	22.9	513.3038	1025.6	0.2
3	2	93.6	7.11E+05	LVNELTEFAK	30.67	582.3184	1163.631	-2.0
4	2	95.6	9.45E+04	DFAEDKDVCK	18.72	585.2607	1169.515	-1.2
5	2	93.9	2.65E+05	VTDLTKVH	22.9	456.7617	912.515	0.1
6	3	93.8	5.60E+05	DEFKADEKKFW	27.07	481.5697	1442.696	-1.5
7	2	86.7	1.19E+05	SHKDDSPDLPK	14.67	619.8041	1238.602	-1.1
8	2	93.7	6.61E+05	TVFDKLK	22.93	425.7576	850.504	4.2
9	2	92.2	5.95E+05	HLVDEPQNLIK	25.33	653.3626	1305.717	0.6
10	3	91.6	2.31E+05	DLGEEHFKGL	27.12	382.1926	1144.564	-1.6
11	2	87.5	3.55E+05	AFDEKLFTF	38.68	559.2813	1117.557	-1.9
12	2	87.0	1.79E+05	TDLTKVH	22.9	407.2273	813.447	-0.3
13	2	78.9	8.78E+05	ADEKKFW	23.7	462.2361	923.463	1.8

14	2	93.3	1.95E+05	DLGEEHFK	19.13	487.7329	974.458	-0.3
15	4	82.6	3.36E+05	RFKDLGEEHFK	19.18	352.1871	1405.723	1.5
16	2	88.6	1.58E+05	GEEHFK	19.13	373.6774	746.347	-0.4
17	2	90.6	2.06E+06	AFDEKLF	31.77	435.2249	869.441	1.3
18	3	91.7	4.24E+05	CADDRADLAK	13.47	359.8392	1077.5	2.1
19	3	60.5	4.97E+05	QQCPFDEHVK	20.78	410.8576	1230.558	-0.4
20	2	96.6	2.27E+05	VSTPTLVEVS	29.63	516.2837	1031.563	-2.8
21	2	91.5	5.92E+06	ALTPDETYVPK	24.42	617.3253	1233.637	4.9
22	2	77.1	3.07E+05	DEKKFW	23.7	426.7165	852.426	-0.4
23	2	91.0	3.45E+05	TKPESERMPCTE	17.18	704.3156	1407.625	-1.0
24	2	89.1	6.17E+05	HEKTPVSE	11.62	463.7326	926.458	-1.0
25	2	94.2	5.36E+06	SALTPDETYVPK	24.88	660.8404	1320.669	3.2
26	2	90.9	1.18E+06	FVEVTK	18.55	361.7107	722.409	6.5
27	2	85.9	6.55E+05	LVNELTEFA	35.23	518.273	1035.536	1.8
28	2	82.7	2.44E+06	NYQEAKDAFL	28.25	599.7932	1198.574	3.4
29	1	97.8	1.02E+06	TPTLVE	23.6	659.3637	659.362	3.3
30	2	90.6	2.64E+06	AEDKGACL	17.4	403.6923	806.372	6.2
31	2	90.6	2.72E+04	KQEPERNECFL	25.33	696.8315	1392.658	-2.1
32	2	96.6	1.93E+05	TVMENFVA	31.15	455.7199	910.434	-2.6
33	3	82.9	4.54E+06	LKPDPNTLCDEFK	29.07	507.2542	1519.747	0.2
34	2	95.5	1.52E+05	ADDRADLAK	13.47	487.749	974.491	-0.4
35	2	88.5	1.53E+05	FDKLK	22.93	325.6975	650.388	-0.9
36	2	69.9	3.13E+05	TALVELLK	33.38	443.7838	886.561	-1.8
37	2	78.5	3.85E+05	ATEEQLK	12.52	409.7168	818.426	-0.2
38	2	91.8	3.00E+06	LTPDETYVPK	22.82	581.8055	1162.6	3.2
39	2	84.0	4.39E+05	QNCDQFEKL	25.63	562.7554	1124.505	-1.4
40	2	60.3	7.02E+05	ECCDKPLL	26.5	460.7141	920.422	-1.9
41	2	92.4	2.13E+06	HLVDEPQN	16.12	476.2304	951.454	-0.7
42	2	94.6	2.29E+05	YICDNQDTIS	23.37	586.2507	1171.494	-0.5
43	2	87.6	3.81E+05	LVNELTE	24.3	409.2198	817.431	1.4
44	2	91.5	2.38E+06	AEFVEVTK	22.57	461.7513	922.489	6.7
45	2	90.2	1.40E+06	DELCKVA	21.52	389.1939	777.382	-2.2
46	2	90.9	4.16E+05	VSTPTLVE	27.25	423.2342	845.462	-1.8

47	2	89.4	3.38E+04	TKPESERMPCTEDYLS	25.28	943.4176	1885.831	-2.0
48	2	87.3	3.21E+05	VEGPKLVV	27.78	420.7625	840.519	-2.7
49	2	92.3	1.37E+05	ECCHGDLLE	23.77	509.7019	1018.397	-1.4
50	2	86.0	2.80E+05	ADFAEDKDVC	21.87	556.7314	1112.457	-1.8
51	2	92.9	5.75E+06	AFDEKLFT	30.55	485.7468	970.489	-2.9
52	3	69.6	1.50E+05	SHKDDSPDLPKL	24.33	451.2332	1351.686	-1.3
53	2	90.1	4.16E+05	YICDNQDTISS	22.75	629.7652	1258.526	-2.9
54	2	78.4	3.54E+05	RHPEYAVS	14.57	479.7411	958.475	-0.4
55	2	76.1	1.09E+05	YANKYN	10.97	386.6844	772.363	-2.5
56	2	94.7	4.16E+05	TPTLVEVS	27.25	423.2342	845.462	-1.8
57	2	65.4	2.12E+06	KLGEYGFQ	25.5	471.2385	941.473	-4.3
58	2	76.7	6.06E+05	VEGPKLVVS	24.97	464.2783	927.552	-2.9
59	2	69.7	1.47E+06	ECCDKPLLE	24.65	525.2363	1049.465	0.0
60	2	59.6	5.71E+04	LAKEYEAT	15.47	462.7371	924.468	-1.6
61	2	76.6	1.08E+05	GKYLYE	21.08	386.6983	772.388	0.9
62	1	84.6	3.34E+06	IAFS	23.33	437.2429	437.24	6.5
63	2	85.5	3.96E+06	EPQNLIK	19.78	421.2447	841.478	3.8
64	2	76.8	2.63E+06	LKPDPNTLC	23.43	500.7599	1000.514	-1.7
65	1	70.0	1.25E+06	RHPYF	18.9	719.3636	719.363	0.9
66	2	84.3	1.24E+06	EAKDAFL	26.02	397.2092	793.41	1.2
67	2	77.0	3.15E+06	RKVPQVS	12.48	407.254	813.495	6.7
68	2	60.9	5.05E+04	AADDKEACFA	20.92	520.7208	1040.436	-2.1
69	1	100	1.67E+05	FVEVT	23.27	594.3132	594.314	-1.2
70	2	38.6	2.08E+04	LVNELTEFAKT	31.35	632.8416	1264.679	-2.9
71	1	89.6	8.25E+05	AWSVA	25.73	533.2725	533.272	0.2
72	3	32.1	1.53E+05	QCPFDEHVK	20.67	368.1715	1102.499	-0.3
73	2	67.1	4.22E+04	EKLFTF	33.92	392.7149	784.424	-3.2
74	2	63.1	2.74E+05	KDDPHACYS	12.43	518.2128	1035.421	-2.6
75	3	59.9	4.19E+04	VEKDAIPENLPPLT	31.68	512.6149	1535.832	-1.9
76	2	73.7	1.80E+05	RHPYFY	22.12	441.716	882.426	-2.4
77	2	72.0	2.74E+05	VADESHAGCE	11.33	509.2005	1017.395	-1.5
78	2	75.1	5.98E+05	SLHTLFG	31.3	387.7104	774.415	-2.5
79	2	47.7	2.00E+05	FHADICT	23.5	403.6792	806.351	-0.1

80	2	88.2	3.15E+05	YEIAR	16.77	326.1775	651.347	0.8
81	1	60.9	1.57E+05	LVTDLTK	21.4	789.4713	789.472	-1.1
82	2	59.2	8.20E+04	NYQEAKDAFLG	26.75	628.3006	1255.596	-2.0
83	2	41.4	1.68E+05	LTKVH	6.92	299.1896	597.372	-1.7
84	1	77.7	3.22E+05	SIQKFG	18.4	679.3785	679.378	0.9
85	2	52.2	4.52E+05	QTALVELLK	33.92	507.8126	1014.62	-2.5
86	1	81.3	3.04E+05	GVFQ	21.1	450.2357	450.235	1.1
87	2	64.4	1.52E+06	CADDRADLA	16.13	475.2057	949.405	-1.3
88	2	69.7	9.52E+04	FVDKCC	18.2	357.6505	714.295	-3.3
89	2	88.5	5.89E+05	RHPEYA	9.75	386.6904	772.374	-1.5
90	2	83.6	1.55E+05	ILNRLC	24.55	366.2152	731.424	-1.7
91	1	61.8	7.73E+05	ENFVA	22.67	579.2782	579.278	0.6
92	1	100	9.40E+05	GSFLYE	29.63	715.3307	715.33	0.6
93	1	86.7	1.29E+05	YAVS	14.8	439.2189	439.219	-0.8
94	2	40.2	4.11E+04	DLGEEHFKG	19.52	516.2425	1031.48	-2.6
95	3	37.4	1.61E+05	LKPDPNTLCDEFKAD	29.47	569.2738	1705.811	-3.0
96	3	66.7	1.14E+05	PFDEHVK	19.32	291.149	871.431	0.1
97	3	38.4	2.20E+05	LSHKDDSPDLPK	16.02	451.2341	1351.686	0.6
98	2	41.9	7.65E+06	LPKIETM	26.03	416.2355	831.465	-2.3
99	2	37.6	8.41E+05	RSLGKVG	12.17	358.7247	716.442	-0.4
100	2	73.9	3.68E+06	PKIETMR	23.28	437.7442	874.482	-1.7
101	2	43.3	1.17E+05	GEEHFKGL	27.2	458.7294	916.453	-2.2
102	2	59.2	4.88E+04	IETMR	14.23	325.1702	649.334	-2.7
103	3	30.7	4.54E+06	HKDDSPDLPK	14.52	384.5279	1151.57	-1.5
104	3	46.7	4.94E+05	DTHKSEIAH	6.32	346.5058	1037.502	0.3
105	2	80.9	6.55E+06	HEKTPVS	9.62	399.2109	797.416	-2.3
106	2	45.9	5.69E+04	KLKECCD	11.38	419.6933	838.38	-1.8

Table S 11. Summary of identified peptide using free papain.

No	Precursor charge	SPI (%)	Intensity	Sequence	RT (min)	Exp. *m/z*	Theor. *m/z*	Error (ppm)
1	2	88.1	3.06E+06	AEFVEVTK	22.52	461.7485	922.489	0.7
2	2	93.2	1.58E+05	LVNELTE	24.27	409.2196	817.431	0.8
3	2	83.5	6.34E+05	AFDEKLFT	30.5	485.749	970.489	1.6
4	2	81.2	4.39E+04	AFDEKLF	31.68	435.2247	869.441	0.8
5	2	94.2	1.23E+06	FVEVTK	18.57	361.7086	722.409	0.7
6	3	85.1	1.54E+05	DLGEEHFKGL	27.08	382.1938	1144.564	1.7
7	2	72.1	1.06E+04	LTPDETYVPK	22.78	581.8042	1162.6	0.9
8	2	81	1.51E+05	GKYLYE	20.95	386.6983	772.388	1
9	2	78.6	1.37E+05	YEIAR	16.72	326.177	651.347	-0.6
10	2	77.1	6.45E+05	ALTPDETYVPK	24.37	617.3233	1233.637	1.7
11	2	58.5	3.68E+04	IQKFG	16.88	296.6771	592.346	0.8
12	2	46.5	2.49E+05	ADEKKFW	23.65	462.2359	923.463	1.5
13	3	50.5	3.44E+05	DTHKSEIAH	5.85	346.506	1037.502	0.8

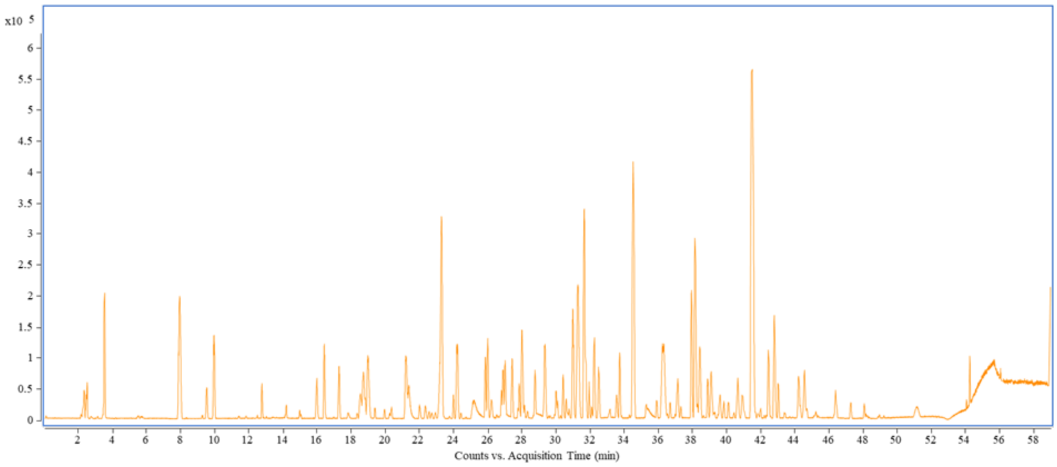

Figure S10. Base peak chromatogram (BPC) of casein digested with free papain.

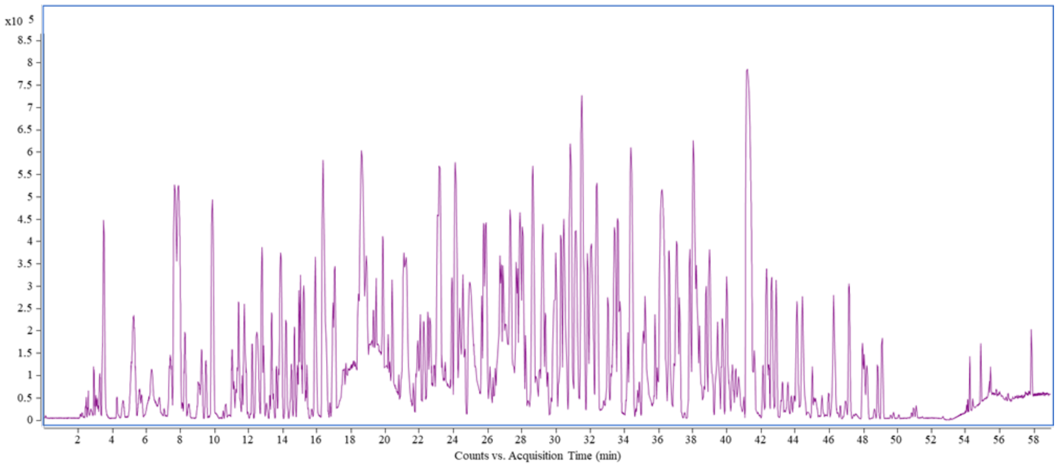

Figure S11. Base peak chromatogram (BPC) of casein digested with SPs-Pap-ATS (10X).

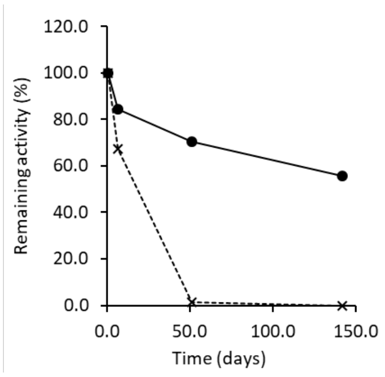

Figure S12. Study of the stability overtime of SPs-Pap-ATS (10X) (solid line) and soluble papain (dashed line) stored at 4°C in phosphate buffer (10 mM, pH 8).

Table S 12. Protein quantification assay performed on reaction supernatant after subtilisin A immobilisation. An average concentration of 64 µg/mL of immobilised subtilisin A was calculated.

OD	$[SubA]_{supernatant}$ (µg/mL)	$[SubA]_{initial}$ (µg/mL)	$[SubA]_{immobilised}$ (µg/mL)
0.2217	201.5	263.6	62.0
0.2218	201.6	263.6	61.9
0.216	196.4	263.6	67.2

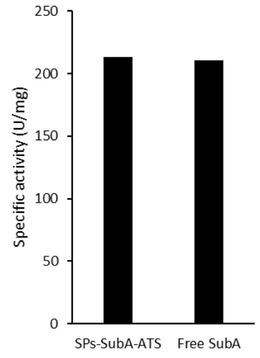

Figure S13. Universal protease assay of both soluble and immobilised SubA using casein as substrate.

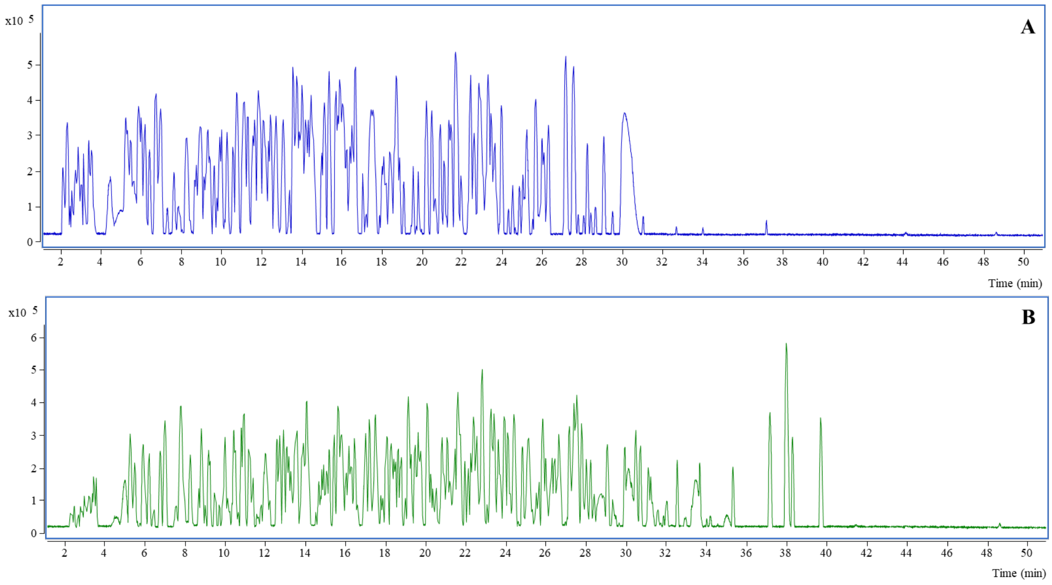

Figure S14. Base peak chromatogram (BPC) of BSA digested with (A) free SubA and (B) SPs-SubA-ATS (10X).

Table S 13. Summary of identified peptide using free SubA.

No	Precursor charge	SPI (%)	Intensity	Sequence	RT (min)	Exp. *m/z*	Theor. m/z	Error (ppm)
1	2	95.6	1.25E+06	DDSPDLPK	18.37	443.7172	886.416	12.3
2	2	94	9.95E+05	KDDSPDLPK	15.98	507.764	1014.511	9.3
3	2	93.5	8.32E+05	VEGPKLV	23.02	371.2331	741.451	9.9
4	2	88.6	1.93E+05	EKKFWGK	16.47	461.7641	922.515	5.8
5	1	96.3	7.11E+05	TPTLVE	23.17	659.3661	659.362	6.9
6	2	87.5	2.59E+05	VEGPKLVV	27.33	420.7654	840.519	4

7	2	90.3	2.55E+05	KFWGKY	22.7	414.7268	828.441	6
8	2	88.8	4.28E+05	DFAEDK	13.88	362.6647	724.315	8.7
9	2	92.4	7.17E+05	VEGPKL	20.63	321.698	642.383	8.5
10	2	88.2	3.02E+05	KTPVSEK	3.57	394.7328	788.452	7.6
11	1	92.3	1.05E+05	AEFVE	21.73	594.2799	594.278	4
12	2	83.5	7.18E+05	DSPDLPK	17.43	386.2013	771.389	7.7
13	2	84.3	4.85E+05	KFWGK	18.45	333.1954	665.378	8.3
14	2	85.8	7.20E+05	KHKPK	2.08	319.2139	637.415	7.7
15	1	88.8	1.06E+05	LFGDE	23.68	580.2636	580.262	2.9
16	2	85.8	1.35E+06	LKPDPN	11.55	342.1949	683.373	13.5
17	1	80.7	3.57E+06	FYAPE	22.38	626.2845	626.283	3
18	2	84.7	7.29E+04	AFDEK	11.65	305.1496	609.288	4.9
19	2	87.9	3.76E+05	VDEPQNL	21.08	407.7037	814.395	6
20	1	83.6	3.23E+05	KYNGVF	23.7	727.3818	727.378	5.3
21	2	90.2	2.75E+05	KFGER	8.8	318.6795	636.347	6.5
22	2	83.5	1.01E+06	TYVPK	14.18	304.1803	607.346	12
23	1	88.6	5.19E+05	KDAFLG	22.28	650.3546	650.351	5
24	2	77.8	1.07E+05	SQKFPKA	14.25	403.2345	805.457	4.9
25	1	84.6	3.68E+05	NGVFQ	21.13	564.2811	564.278	5.2
26	2	87.9	3.88E+05	GKVGTR	3.43	309.1938	617.374	10.2
27	2	85	5.96E+04	TVFDKL	27.5	361.7102	722.409	5.1
28	1	87.5	5.39E+05	TADFA	18.6	524.2388	524.236	5.9
29	1	89	1.64E+05	EGPKLV	23.02	642.3848	642.383	3.4
30	2	73	2.15E+06	HFKGL	17.58	301.1804	601.346	11.3
31	1	89.5	5.27E+05	EGPKL	20.63	543.3173	543.314	5.6
32	2	85	3.77E+05	TRKVPQVS	13.13	457.7785	914.542	7.5
33	1	80.8	5.29E+05	IETM	18.18	493.2367	493.233	7.1
34	2	53.6	9.23E+05	EKKFW	21.92	369.2067	737.399	9.4
35	1	80.4	6.25E+05	YGFQ	21.35	514.2331	514.23	5.6
36	1	83	9.57E+05	VEGPK	5.87	529.3007	529.299	4
37	2	93.6	2.21E+05	IVRY	16.22	275.6731	550.335	5.4
38	2	62.5	1.04E+05	LKPDPNTL	21.27	449.2581	897.505	4.3
39	1	81.8	8.01E+04	VTDLT	17.08	548.295	548.293	3.3
40	2	68.9	1.59E+05	FAEDK	10.13	305.1497	609.288	5.2

41	1	83.4	1.52E+06	FGDE	13.73	467.1803	467.178	5.3
42	1	82.1	9.43E+05	KTVM	11.5	478.2741	478.27	8.7
43	2	75.7	9.60E+05	RKVPQVS	12.18	407.2561	813.495	11.9
44	1	79.7	3.19E+06	TVFD	20.45	481.2375	481.23	15.9
45	1	77.8	6.15E+05	GVFQ	20.72	450.2374	450.235	4.7
46	1	81.2	2.05E+06	VSTPT	13.03	504.2707	504.267	7.4
47	1	74.5	3.23E+06	VADE	4.88	433.1957	433.193	5.2
48	1	76.7	1.31E+05	DNQDTIS	13.12	792.3412	792.338	4.6
49	1	68.4	1.16E+06	FKAD	10.25	480.2506	480.246	10
50	1	77.2	8.55E+05	AIPE	15.93	429.2389	429.235	9.2
51	1	74.5	4.53E+05	QDTISS	12.48	650.3014	650.3	2.6
52	2	63.8	2.85E+05	CDKPL	18.13	288.1481	575.286	3.8
53	2	41.6	1.65E+04	FWGKY	25.77	350.6782	700.346	3.8
54	2	74.5	5.43E+04	SARQR	2.35	309.1794	617.348	4.3
55	2	48.9	7.95E+05	DEHVKL	17.85	370.7035	740.394	6.7
56	2	51.7	2.77E+05	CTKPE	10.37	289.1378	577.266	3.8
57	1	47.9	3.59E+05	GDMA	8.97	393.1466	393.144	5.7
58	1	63.7	2.18E+05	ANKY	5.62	495.2582	495.257	2.9
59	2	55.8	2.74E+05	KFPKA	13.68	295.6885	590.367	4.4

Table S 14. Summary of identified peptide using SPs-SubA-ATS (10X).

No	Precursor charge	SPI (%)	Intensity	Sequence	RT (min)	Exp. *m/z*	Theor. m/z	Error (ppm)
1	2	92	1.89E+05	YICDNQDTISSK	20.25	693.818	1386.621	5.1
2	2	97.1	3.36E+05	KDDSPDLPKLKPDPNTL	26.52	947.0078	1892.997	5.7
3	2	98.2	2.94E+04	DDSPDLPKLKPDPNTL	28.72	882.9591	1764.902	4.7
4	3	94.2	1.22E+06	KDLGEEHFKGL	22.37	424.8972	1272.659	13.5
5	3	85.6	3.05E+05	DTHKSEIAHRF	14.8	447.5662	1340.671	8.7
6	2	96.2	3.80E+05	KYICDNQDTISSK	17.98	757.8673	1514.716	7.1
7	2	98	2.56E+05	AEDKDVCKNY	22.2	592.7699	1184.526	5.2
8	2	95.7	2.96E+05	ICDNQDTISSK	15.75	612.2867	1223.558	6.4
9	2	98.3	1.67E+05	PDTEKQIK	16.95	479.7674	958.521	6.2
10	2	100	6.29E+04	TADFAEDKDVCKNY	22.4	809.8609	1618.706	5
11	2	93.2	3.83E+05	LFGDELCKVA	32.95	547.7847	1094.556	5.4
12	2	97.2	1.30E+05	FAEDKDVCKNY	19.6	666.3045	1331.594	5.2
13	2	97.8	1.27E+05	VADESHAGC	9.72	444.6822	888.352	5
14	2	96.8	1.15E+06	VADESHAGCE	10.3	509.2048	1017.395	7
15	2	94.6	3.32E+05	VDEPQNLIKQ	23.23	592.3235	1183.632	5.9
16	2	97.2	3.28E+05	PDTEKQIKKQ	14.87	607.8448	1214.675	6.1
17	2	92.8	2.59E+06	VDEPQNLIK	23.6	528.2974	1055.574	12.6
18	2	94.4	1.73E+05	VTDLTKVH	16.57	456.7645	912.515	6.3
19	3	91.3	2.19E+05	DFAEDKDVCK	18.32	390.5134	1169.515	8.4
20	2	95.7	1.98E+06	DFAEDKDVCKNY	22.2	723.8221	1446.621	10.5
21	3	94.3	9.28E+04	AFDEKLFTFH	32.7	418.8797	1254.616	6.1
22	2	94.6	2.49E+05	CDKPLLEK	17.08	473.2612	945.508	7
23	2	89.8	4.91E+05	TLPDTEKQIKKQ	14.87	714.9121	1428.806	7.1
24	2	87	1.93E+05	CPFDEHVKL	27.23	544.2691	1087.525	5.4
25	2	94.5	8.63E+05	FGDELCKVA	27.4	491.2467	981.471	14.4
26	3	89.8	2.07E+06	LQQCPFDEHVKL	29.45	486.2513	1456.726	8.5
27	4	93	4.82E+04	ICTLPDTEKQIKKQ	20.12	411.984	1644.9	8
28	2	92.9	8.96E+05	AFDEKLF	31.25	435.2275	869.441	7.3
29	3	97.6	1.24E+05	KHLVDEPQNLIKQ	22.02	521.2982	1561.87	5.6

30	2	92.2	6.41E+04	KHLVDEPQNLIK	22.1	717.4135	1433.812	5.2
31	2	94.1	3.68E+05	GEEHFKGL	22.38	458.7337	916.453	7.3
32	2	96.4	2.47E+04	DKGACLLPKIE	29.03	593.8316	1186.651	4.1
33	2	91.7	6.87E+04	FDEHVKL	27.22	444.2379	887.463	5.9
34	2	94.9	2.43E+05	PFDEHVKL	27.32	492.7641	984.515	4.9
35	2	91.2	1.40E+05	HEKTPVSEK	3.55	527.7834	1054.553	5.4
36	2	89.3	3.40E+04	DTEKQIKKQ	14.88	559.3174	1117.622	4.8
37	4	92.6	2.81E+05	HKDDSPDLPKLKPDPN	20.62	454.7406	1815.924	8.2
38	2	98.1	1.33E+05	YTRKVPQVSTPTLVE	25.23	859.4831	1717.949	5.6
39	3	94	2.59E+05	TLPDTEKQIK	16.93	391.5594	1172.653	8.3
40	3	79.2	6.55E+04	LQQCPFDEHVKLVN	28.62	557.2881	1669.837	6.8
41	2	96	1.89E+05	EKKFWGKY	20.38	543.2964	1085.578	6
42	2	91.8	8.70E+05	EDKGACLLPKIE	28.85	658.3545	1315.693	6.2
43	2	98.1	1.21E+06	ECADDRADLA	17.18	539.7341	1078.448	11.9
44	2	98.4	2.99E+06	VEGPKLVVS	24.35	464.2829	927.552	7
45	3	87.8	1.20E+05	CVLHEKTPVSEK	15.68	457.2465	1369.715	6.6
46	2	92.9	2.36E+05	HEKTPVSEKVTKC	12.05	743.3946	1485.774	5.3
47	2	84.3	7.42E+05	CDEFKAD	16.25	414.1703	827.324	10
48	3	83.5	7.37E+04	CVLHEKTPVSEKVTKC	17.52	600.9875	1800.935	6.5
49	4	88.2	4.82E+04	VLHEKTPVSEKVTKC	15.87	425.2408	1697.926	8
50	2	93.2	1.01E+06	YSTVFDKL	31.22	486.7601	972.504	8.4
51	2	88.2	2.43E+05	CDKPLLEKS	16.88	516.7767	1032.54	5.4
52	2	85.2	9.05E+05	EDKGACLLPKIETM	31.98	774.399	1547.781	5.7
53	2	95	2.25E+05	HEKTPVSE	10.55	463.7357	926.458	5.6
54	2	93	2.13E+05	EEHFKGL	20.32	430.2206	859.431	2.4
55	2	86	2.48E+05	TDLTKVH	16.57	407.2306	813.447	7.9
56	2	94.7	9.26E+05	CCDKPLLE	23.03	460.718	920.422	6.5
57	2	95.2	6.34E+05	VEKDAIPENLPPLTA	31.65	803.943	1606.869	5.6
58	2	93	1.80E+05	ECCDKPLLE	24.15	525.2394	1049.465	6
59	2	97.9	5.37E+05	AVEGPKLVVS	24.87	499.8012	998.589	6
60	2	95.5	3.92E+05	VEGPKLVV	27.3	420.7667	840.519	7.3
61	2	81.6	1.42E+05	EDKGACLLPK	21.6	537.2902	1073.566	5.6
62	2	88	5.04E+05	QCPFDEHVKL	27.97	608.2997	1215.583	7
63	2	86.4	9.13E+04	DKPLLEKS	16.88	465.2721	929.531	5.9

64	2	97.7	3.87E+05	YTRKVPQVSTPTLVEVS	27.27	952.5348	1904.049	6.5
65	3	78.5	7.17E+05	QCPFDEHVKLVN	27.23	476.908	1428.695	9.6
66	3	88.8	3.96E+05	HEKTPVSEKVT	12.18	418.898	1254.669	7.1
67	2	92.7	1.02E+06	LLPKIETM	29.4	472.7815	944.549	6.5
68	2	98.8	1.01E+06	KVPQVSTPTLVEVS	29.28	742.4282	1483.837	7.7
69	2	98.2	1.09E+06	RKVPQVSTPTLVEVS	26.37	820.4794	1639.938	7.7
70	2	93	4.56E+05	VEGPKLV	23.02	371.2312	741.451	4.8
71	2	86	9.05E+04	DKPLLEK	17.08	421.7557	842.499	5.8
72	2	91.3	1.06E+06	TPDETYVPK	19.15	525.2647	1049.516	5.8
73	2	92.6	1.25E+06	ALTPDETYVPK	23.97	617.3274	1233.637	8.4
74	2	95.8	1.11E+06	CADDRADLA	15.95	475.2103	949.405	8.3
75	2	86.1	6.87E+05	EIAHRF	16.22	386.7132	772.411	10.4
76	2	84.8	2.86E+05	EDKDVCKNY	13.98	557.252	1113.489	6.8
77	2	95.1	2.42E+05	KECCHGDLL	20.93	509.2316	1017.45	5.6
78	1	100	9.48E+05	AFDEKLFTF	38.25	1117.565	1117.557	6.8
79	2	92.8	5.45E+05	GACLLPKIE	31.05	472.2713	943.529	6.4
80	2	92.1	9.90E+05	KLVTDLTK	19.4	459.2925	917.567	10.9
81	2	93.9	3.41E+05	LIVRYT	22.63	382.7403	764.467	7.5
82	3	82.3	4.76E+04	LKECCDKPLLEKS	19.98	502.5985	1505.771	6
83	2	86.7	2.49E+05	GDELCKVA	21.3	417.7083	834.403	6.8
84	3	75.1	2.41E+05	QQCPFDEHVKLVN	27.08	519.5932	1556.753	6.9
85	2	83.2	6.34E+05	SQKFPKAE	13.37	467.7577	934.5	8.2
86	2	88.8	1.18E+06	SLRETYGDM	21.92	536.2486	1071.478	10.5
87	2	97.7	4.09E+05	TRKVPQVSTPTLVEVS	26.67	871.0025	1740.986	6.5
88	2	87.1	4.78E+05	TEFAKTC	16.5	400.1897	799.366	7
89	1	96.5	4.91E+05	EFVEVT	25.62	723.361	723.356	6.2
90	3	88	6.72E+05	RLSQKFPKAE	15.77	401.9042	1203.685	10
91	2	84.2	1.41E+05	DTHKS	2.32	294.1451	587.279	5.7
92	2	91.5	1.43E+06	CDKPLLE	21.68	409.2169	817.413	16
93	3	72.5	3.21E+05	QQCPFDEHVKL	27.82	448.5557	1343.642	7.2
94	2	96.3	1.08E+05	TRKVPQVSTPTLVE	24.45	777.9505	1554.886	4.9
95	2	85.2	2.02E+05	NCDQFEKL	25.2	498.7296	996.446	5.4
96	3	89.2	6.36E+05	RSLGKVGTR	9.98	325.206	973.591	11.8
97	3	70.8	1.08E+05	QAEDKGACLLPKIE	28.62	505.6044	1514.789	5.8

98	2	95.5	6.64E+05	ADDRADLA	14.77	423.7055	846.396	8.9
99	3	90.3	5.40E+04	CDKPLLEKSHCIA	21	486.2511	1456.729	5.8
100	2	83.4	9.17E+05	DNQDTISSK	10.47	504.2416	1007.465	10.8
101	3	84	9.95E+04	HKDDSPDLPK	13.93	384.5311	1151.57	6.8
102	2	87.7	3.58E+05	EKKFWGK	16.3	461.765	922.515	7.7
103	2	91.5	4.29E+05	LTPDETYVPK	22.35	581.8076	1162.6	6.7
104	2	86.8	1.57E+07	AIPENLPPLTA	33.45	568.3272	1135.636	9
105	2	85.7	3.76E+04	LKECCDKPLLE	23.68	645.8282	1290.644	3.7
106	2	95.2	3.38E+05	KVPQVSTPTLVE	27.1	649.376	1297.737	5.7
107	1	98.1	3.83E+06	NLPPLTA	28.68	725.4246	725.42	6.6
108	3	78.2	5.22E+05	CPFDEHVKLVN	26.5	434.2218	1300.636	10.6
109	3	96.3	1.21E+05	TESLVNRRPCFSAL	29.28	531.616	1592.822	6.5
110	3	96.3	2.22E+05	LYEYSRRHPEYAVS	19.5	590.6297	1769.861	6.9
111	2	84	1.65E+05	CCTKPESE	9.27	448.6807	896.349	4.6
112	2	96.3	1.25E+05	VEKDAIPENLPPL	32.82	717.8994	1434.784	4.6
113	2	92.6	4.26E+05	RKVPQVSTPTLVE	24.05	727.4263	1453.838	4.8
114	2	94	3.16E+05	SVARLS	14.13	316.693	632.373	7.9
115	2	87.3	2.18E+05	KFWGKY	22.28	414.7263	828.441	4.7
116	2	90.4	5.72E+05	KHLVDEPQNL	20.8	596.8234	1192.633	5.4
117	2	92.5	6.62E+05	SERMPCTEDYL	28.47	672.288	1343.561	5.2
118	2	88.8	5.90E+05	SLVNRRPCFS	21.92	589.8138	1178.61	7.9
119	3	71.4	2.53E+04	CPFDEHVKLV	28.63	396.205	1186.593	5.3
120	2	92.4	7.60E+04	GEYGFQN	21.42	407.6751	814.337	6.3
121	1	90.7	2.81E+05	FGDELC	26.77	683.2738	683.271	4.1
122	3	76.7	2.11E+04	KDDSPDLPKLKPDPN	22.05	560.2972	1678.865	6.4
123	2	84.9	2.73E+04	TKPESERMPCTEDYLSL	31.33	999.9661	1998.915	4.5
124	2	90.6	3.84E+05	LKPDPNTL	21.23	449.2591	897.505	6.4
125	2	86.3	2.31E+04	ANKYNGVFQEC	25.48	636.7905	1272.568	3.8
126	2	95.7	5.31E+06	SERMPCTEDYLSL	33.63	772.35	1543.677	9.6
127	2	92.6	9.70E+04	LEECCAK	13.82	398.1753	795.338	6
128	2	88.8	2.50E+05	KYNGVFQE	22.45	492.7463	984.479	5.8
129	2	85.4	6.37E+05	KSLHTL	13.95	349.7176	698.42	10.3
130	3	83.1	2.67E+04	QAEDKGACLLPKIETM	31.68	582.9677	1746.877	6
131	2	86	1.58E+06	STVFDKL	28.27	405.2263	809.441	4.7

132	1	88.5	1.32E+06	NCDQFE	18.73	755.2731	755.267	8
133	2	86.3	1.30E+05	DFAEDK	13.37	362.6638	724.315	6.1
134	2	82.1	5.84E+05	VNRRPCFS	15.75	489.7553	978.494	8.5
135	4	70.1	6.38E+04	HEKTPVSEKVTK	10.02	346.4497	1382.764	8
136	2	85.8	2.94E+05	KYNGVFQEC	25.27	544.251	1087.488	5.5
137	1	90.2	2.90E+05	LFGDEL	32.88	693.3483	693.346	3.4
138	2	90.3	3.97E+05	KFGERAL	18.1	410.7407	820.468	6.8
139	2	94.3	5.73E+05	IVRYT	15.03	326.1988	651.383	10.4
140	2	91.8	1.62E+05	QKFPKAEFVEVT	26.5	711.8893	1422.763	5.2
141	2	89.3	1.23E+06	VTDLTK	12.87	338.7033	676.388	15.6
142	2	86	1.03E+06	SLVNRRPCFSAL	28.37	681.8747	1362.732	7.3
143	2	93.4	3.23E+06	RMPCTEDYLSL	33.52	664.3103	1327.603	7.6
144	2	86.5	1.43E+05	EEQLKTV	17.58	423.735	846.457	5.9
145	2	91	7.90E+04	AKEYEATLE	19.68	527.2619	1053.51	5.3
146	2	70.6	6.92E+04	NQDTISSK	9.45	446.7253	892.438	5.8
147	2	95.4	1.22E+05	KEYEATLE	19.2	491.7434	982.473	5.7
148	3	92	8.22E+04	ARLSQKFPKAE	16.75	425.5822	1274.722	6.9
149	2	92	3.68E+05	SRRHPEYAVS	11.75	601.3115	1201.608	6.2
150	2	90.3	7.62E+06	AIPENLPPL	35	482.2825	963.552	5.9
151	2	79.8	4.37E+04	EEQLKTVM	22.7	489.2551	977.498	4.7
152	2	89.8	1.06E+05	LVTDLTK	20.9	395.2422	789.472	5.5
153	2	86.9	2.53E+05	KFWGK	18.38	333.1948	665.378	6.4
154	3	84.2	1.43E+06	DSPDLPKLKPDPNTL	28.28	550.6341	1649.875	7
155	1	93.7	5.86E+05	TPTLVE	23.12	659.3648	659.362	4.9
156	2	85.2	6.45E+04	KSLHTLF	25.18	423.2498	845.489	3.8
157	2	89.6	2.17E+05	KVPQVSTPT	18.77	478.7771	956.542	5
158	2	83.9	6.13E+05	EYSRRHPEYAVS	14.73	747.3662	1493.714	7.3
159	2	91	5.44E+04	AFDEK	10.95	305.1495	609.288	4.6
160	2	86.1	1.09E+06	TVFDKL	27.5	361.7134	722.409	14
161	2	88.8	5.37E+05	EVEKDAIPE	18.95	515.2624	1029.51	6.4
162	2	81.8	3.63E+05	QEAKDAFLGS	22.87	533.2686	1065.522	7.2
163	1	92.8	2.30E+05	DDKEACFA	20.7	898.3659	898.362	4.7
164	2	91.9	3.99E+05	LIVRY	24.15	332.2166	663.419	8.9
165	2	90	1.28E+06	RRHPYFYAPE	21.18	668.3395	1335.66	8.4

166	1	92.6	3.27E+05	STPTLVEV	27.02	845.4659	845.462	4.5
167	3	83.8	3.07E+04	VADESHAGCEKS	3.6	411.515	1232.522	6.2
168	2	79.2	4.60E+04	EKQEPERNE	3.55	579.7762	1158.539	4.8
169	2	86.3	1.40E+06	VNRRPCFSAL	25	581.817	1162.616	9.1
170	3	95.2	1.83E+05	RLSQKFPKAEFVEVT	26.13	593.6691	1778.98	6.2
171	2	75.5	1.05E+05	SERMPCTE	17	476.6998	952.387	5.1
172	4	93.8	2.71E+04	EIARRHPYFYAPELL	32.7	469.5077	1874.992	8.3
173	1	90.3	7.34E+05	VNELTEF	30.62	851.4204	851.415	6.2
174	3	80.1	8.77E+04	LPDTEKQIKKQ	14.87	443.2613	1327.758	7.4
175	2	87.1	2.28E+05	AKEYEAT	10.2	406.1981	811.384	5.7
176	2	78.7	1.71E+05	VHKECCHGDLL	18.93	627.2959	1253.577	5.4
177	2	90.2	9.67E+05	VFDKL	24.05	311.1881	621.361	11.6
178	2	52.5	3.27E+05	FGDELCK	21.73	406.1901	811.366	7.9
179	3	93.1	1.54E+05	EIARRHPYFYAPE	22.9	550.2824	1648.824	4.9
180	2	93.4	8.59E+04	CCHGDLLE	22.3	445.184	889.355	6.1
181	1	90.6	3.18E+05	TEDYLSL	32.93	840.4032	840.399	4.9
182	2	90.7	8.58E+05	ALIVRY	25.98	367.7356	734.457	9.4
183	2	85.5	7.17E+04	HTLFGDEL	31.68	466.2324	931.452	4.7
184	2	92.6	3.83E+05	DDRADLA	14.95	388.1866	775.359	8.6
185	2	63.7	4.97E+04	AEDKDVCK	18.32	454.216	907.419	5.3
186	2	83	2.37E+05	TYVPK	13.77	304.1788	607.346	7
187	2	77.4	8.16E+04	EHFKGL	20.32	365.7004	730.389	5.8
188	1	81.3	3.36E+06	ELTEF	27.72	638.3064	638.304	4.2
189	2	60.5	2.06E+05	CDQFEKL	24.2	441.708	882.403	5.7
190	3	48.7	4.93E+05	QCPFDEHVKLV	29.33	438.8923	1314.652	7.3
191	3	81.6	6.85E+05	DKGACLLPKIETM	32.22	473.5881	1418.739	7.1
192	3	76.3	7.83E+05	KTPVSEKVTKC	12.55	407.2324	1219.672	7.9
193	2	72.8	3.40E+05	QKFPKAE	12.93	424.2404	847.468	6.2
194	3	95.2	1.79E+06	RRHPYFYAPELL	31.57	521.2851	1561.828	7.6
195	1	85.8	1.08E+06	TALVE	19.58	532.3046	532.298	11.9
196	2	85	2.15E+06	DEKLFTF	35.28	450.2341	899.452	9.8
197	2	69.1	4.91E+05	SLRETYGDMA	21.92	571.7657	1142.515	7.4
198	2	87.5	8.51E+04	KLVTDL	24.42	344.7179	688.424	5.1
199	4	91.2	4.26E+04	RYTRKVPQVSTPTLVE	23.32	469.2722	1874.05	8.1

200	2	80.7	7.21E+05	VEKDAIPE	17.7	450.7411	900.468	7.3
201	2	87.8	3.48E+05	ALKAW	23.28	294.6817	588.351	7.9
202	2	81.5	7.41E+04	LKPDPN	10.73	342.1916	683.373	3.6
203	2	85.3	8.69E+04	TRKVPQVSTPT	16.87	607.3504	1213.69	2.1
204	2	87.3	1.47E+05	RKVPQVSTPT	16.13	556.8283	1112.643	5.4
205	2	72.9	2.21E+05	RLAKEYEAT	13.3	540.7918	1080.569	6.2
206	1	90.2	2.65E+06	FVEVT	22.78	594.317	594.314	5.3
207	2	75.2	2.04E+05	SQKFPKAEFVEVT	26.65	755.4055	1509.795	5.3
208	2	88.6	2.18E+05	EGPKLVVS	22.28	414.7481	828.483	6.4
209	2	85.7	1.24E+05	SERMPC	16.12	361.6541	722.297	5.2
210	2	75.3	1.36E+05	SARQRLR	3.53	443.7734	886.534	6.1
211	2	73.5	1.92E+06	HFKGL	17.47	301.1808	601.346	12.6
212	2	86	2.38E+05	KYNGVFQ	21.58	428.2245	855.437	5.5
213	2	74.6	2.61E+06	GACLLPKIETM	34.15	588.3159	1175.617	6.1
214	2	84.6	8.89E+04	KEYEAT	9.4	370.6792	740.347	5.2
215	2	74.7	1.71E+06	AIPENLPPLTAD	32.42	625.8377	1250.663	3.5
216	2	75.8	1.82E+06	QEAKDAFLG	23.37	489.7576	978.49	18.1
217	3	65.9	2.02E+04	CQAEDKGACLLPKIE	28.92	539.9415	1617.798	6.7
218	1	80.3	3.17E+06	CDEF	20.2	513.1695	513.165	7.7
219	1	75.4	1.82E+05	TYGDM	18.27	586.2227	586.218	7.6
220	2	77.2	4.55E+05	VDEPQNL	21.03	407.7044	814.395	7.8
221	2	63.1	5.94E+05	VEGPKLVVST	25.07	514.8078	1028.599	8.3
222	2	53.4	3.50E+05	SIQKF	18.37	311.6847	622.356	8.4
223	2	81.9	7.35E+04	GACLLPK	23.28	351.2066	701.402	4.9
224	2	55.8	5.40E+04	CEKSLHTL	17.07	465.7429	930.472	6.5
225	1	86.1	1.50E+06	CTESL	18.22	552.2402	552.234	11.4
226	2	81.7	2.70E+04	RLAKEYEATLE	20.37	661.8552	1322.696	5.3
227	2	91.5	4.65E+05	CCHGDLL	23.7	380.663	760.312	7.9
228	2	87.1	7.93E+05	GKVGTR	3.42	309.1937	617.374	9.7
229	2	70.8	9.08E+04	SQKFPKA	13.87	403.2344	805.457	4.7
230	3	60.6	6.89E+04	KLVTDLTKVH	20.77	385.2392	1153.695	6.6
231	2	79.8	1.45E+05	RETYGDM	17.08	436.1872	871.362	5.4
232	2	52.6	3.62E+04	LRLAKEYEAT	19.72	597.3336	1193.653	5.4
233	1	80.6	1.36E+06	IETM	18.05	493.2397	493.233	13.2

234	3	78.8	2.86E+04	RSLGKVGTRC	11.87	359.5409	1076.6	6.6
235	2	65	1.02E+04	LHTLFGDEL	35.47	522.7745	1044.537	4.5
236	1	89	1.35E+06	YYAN	11.13	530.2305	530.225	10.2
237	1	81.6	9.78E+04	ACFAV	28.68	510.2399	510.239	2.5
238	2	75.1	3.56E+05	RKVPQVS	11.3	407.2543	813.495	7.5
239	2	74.8	7.59E+04	RRPCFS	13.72	383.1972	765.383	4.7
240	2	61	3.91E+04	KTPVSEK	3.55	394.7314	788.452	3.9
241	2	34.1	1.03E+05	QCPFDEH	20.23	438.1733	875.336	3.4
242	2	90.5	1.22E+05	IVRY	16	275.6729	550.335	4.8
243	1	77	6.39E+05	MENF	22.08	540.2165	540.213	6.9
244	2	63.4	1.43E+05	RETYGDMA	17.35	471.7059	942.399	5.2
245	2	79.1	3.07E+05	RRHPEYAVS	11.62	557.795	1114.576	5.7
246	1	85.3	1.67E+05	CVADE	9.92	536.2049	536.203	4.2
247	2	62	1.02E+06	DDPHAC	7.52	329.1208	657.23	5.3
248	1	76.8	1.18E+06	HCIA	9.93	443.2125	443.208	10.9
249	2	52.9	4.19E+04	VAFVDKC	22.93	391.2013	781.392	3.7
250	3	72.3	6.00E+04	SLVNRRPCFSA	23.07	417.2235	1249.648	5.8
251	2	88.7	1.24E+05	TPTLVEVS	27.02	423.2361	845.462	2.8
252	2	62.2	7.52E+05	CCTKPE	7.25	340.6426	680.275	3.9
253	2	56.6	1.32E+06	KDAIPENLPPLTA	31.02	689.8861	1378.758	4.5
254	2	53.3	9.92E+04	KLKEC	3.57	310.6774	620.344	4.4
255	2	67.9	1.19E+05	VNRRPCFSA	19	525.2741	1049.532	8.4
256	2	63.1	4.72E+04	RRPCFSAL	24.1	475.2575	949.504	3.2
257	2	80.1	1.70E+05	SARQR	2.32	309.1803	617.348	7
258	2	62.2	2.30E+05	ALIVRYT	24.52	418.2588	835.504	6.8
259	2	76	2.68E+05	QRLRCA	10.15	373.7109	746.41	5.8
260	2	82.4	6.50E+04	KYNGVF	23.65	364.1944	727.378	4.1
261	1	74.2	2.03E+06	AIPE	15.77	429.2409	429.235	13.9
262	3	34.1	4.41E+05	FAEDKDVCK	15.63	352.1701	1054.488	6.3
263	2	74.7	6.50E+04	YSRRHPEYAVS	13.62	682.843	1364.671	5.2
264	2	53.5	7.07E+04	ICDNQDTISS	17.43	548.2381	1095.463	5.1
265	2	57.4	6.43E+04	DDPHACY	16.15	410.6528	820.294	5
266	1	84	5.93E+05	ADCC	7.03	411.1027	411.101	4.6
267	2	42.5	2.00E+05	TEFAKT	13.93	348.6844	696.357	5.8

268	2	48.7	4.47E+05	LCKVA	14.73	267.1611	533.312	4.3
269	2	35.7	1.07E+05	RHPEYA	9.37	386.6924	772.374	3.7
270	2	51.7	1.69E+06	KDAIPENLPPL	32.23	603.8435	1206.673	4.8
271	1	63.2	5.93E+05	VRYT	15.03	538.3008	538.299	3.5
272	1	65.4	1.37E+06	PHAC	7.52	427.1779	427.176	3.7
273	1	80.4	4.00E+05	CKVA	14.48	420.2298	420.228	4
274	2	32.4	1.49E+05	ATEEQLKTV	18.85	509.7776	1018.542	5.2
275	2	38.9	1.75E+06	KSHCIA	7.72	329.675	658.335	11.4
